# Hydrothermal synthesis and optimization of hierarchical copper molybdate nanostructures for photocatalytic degradation of crystal violet and antimicrobial applications

**DOI:** 10.1038/s41598-025-32124-5

**Published:** 2026-01-13

**Authors:** Rania Hassan, Rabeea D. Abdel‑Rahim, Gamal A. Gouda, Adham M. Nagiub

**Affiliations:** Department of Chemistry, Faculty of Science, Assiut Branch, Al-Azhar University, Assiut, 71524 Egypt

**Keywords:** CuMoO₄, Physical and optical properties, HCM, Photodegradation, Crystal violet dye, Antimicrobial activity, Chemistry, Environmental sciences, Materials science, Nanoscience and technology

## Abstract

In this work, the Hierarchical Copper Molybdate Nanostructures (HCM) photocatalyst was synthesized via a hydrothermal method and characterized using XRD, FTIR, UV-vis spectroscopy, SEM, TEM, and EDX analyses, confirming the formation of hierarchical nanocrystalline structures with a direct band gap of ~ 2.4 eV. The photocatalytic performance of HCM was systematically evaluated for the degradation of crystal violet (CV) dye under UV irradiation. Experimental design and process optimization were conducted using response surface method (RSM) and ANOVA, which demonstrated that pH, irradiation time, and catalyst dosage were the most influential variables, whereas dye concentration exerted a minor effect. Under optimized conditions (pH 10, 60 min, 15 mg/L CV, and 20 mg catalyst dosage), more than 99% CV dye degradation was achieved. Kinetic analysis demonstrated that the degradation followed a pseudo-first-order model, while thermodynamic studies indicated that the process is spontaneous, endothermic, and entropy-driven. Mechanistic evaluation confirmed that reactive oxygen species (·OH, O_2·_, HOO·) generated through electron–hole separation played a dominant role in CV mineralization. In addition, HCM exhibited significant antimicrobial activity against bacterial and fungal strains, supporting its multifunctional potential. Overall, the findings highlight HCM as a highly efficient, low-cost, and environmentally friendly photocatalyst with promising applications in wastewater treatment and environmental remediation.

## Introduction

Water is a major source of human life and other organisms on Earth^1^. The explosive development of the Industrial Revolution and the rise in pollutants in water systems have had a big impact on the purity of water and our environment^2^. Widespread water pollution is caused by these contaminants, which include fertilizers, heavy metals, and organic pollutants. Organic dyes are considered one of the most dangerous industrial wastes and are highly toxic, and hurt the environment^3^. Different remediation mechanisms have been developed to remove such persistent organic pollutants from wastewater. Various methods include coagulation, reverse osmosis, adsorption, chemical oxidation, and biosorption^4–6^. Also, the Fenton process is one of the most important and effective advanced oxidation processes employed for the removal of various organic and inorganic pollutants from water and wastewater^7,8^. It is based on the catalytic reaction between ferrous ions (Fe²⁺) and hydrogen peroxide (H₂O₂) to generate highly reactive hydroxyl radicals (·OH), which possess a strong oxidizing power capable of degrading a wide range of persistent contaminants^9^. Due to its simplicity, high efficiency, and low operational cost, the Fenton process has attracted significant attention in environmental remediation^10^.

Among the above technologies, photocatalysis is a promising and versatile process that utilizes light energy to activate a semiconductor material, leading to the generation of electron-hole pairs capable of driving a variety of redox reactions^11^. This technique has gained considerable attention due to its wide range of environmental and industrial applications^12–15^. Photocatalysis is employed in pollutant degradation, organic synthesis, hydrogen production through water splitting, ammonia production via nitrogen reduction, and photo-inactivation of microorganisms for disinfection purposes^16,17^. The process offers an environmentally friendly and sustainable approach, as it often uses solar energy as the light source and operates under mild conditions^18^.

Copper molybdate (CuMoO₄) is a mixed-metal oxide material that has attracted significant interest in photocatalysis due to its unique electronic structure, redox properties, and visible-light activity. CuMoO₄ has a narrow band gap (≈ 1.3–2.2 eV), allowing it to absorb visible light effectively, unlike classical photocatalysts such as TiO₂, which mainly respond to UV light^19^. This makes CuMoO₄ suitable for solar-driven photocatalysis, increasing its efficiency under sunlight^18^. CuMoO₄ is a mixed-valence oxide containing Cu²⁺/Cu⁺ and Mo⁶⁺/Mo⁵⁺ redox pairs^20,21^. These multiple oxidation states enable internal charge transfer and facilitate electron–hole separation, reducing recombination losses. The Cu and Mo sites act as electron and hole traps, respectively, enhancing photocatalytic lifetime and reactivity^22^. Cu²⁺/Cu⁺ can participate in photo-Fenton-like reactions, producing reactive oxygen species (ROS) such as ·OH and O_2_. Mo^6⁺^/Mo^5⁺^ can act as an electron mediator, further improving the redox capability of the system^23^. This synergistic effect enhances the oxidation of organic pollutants and the reduction of metal ions or CO₂. CuMoO₄ has been successfully used for photodegradation of dyes and organic pollutants (e.g., methylene blue, rhodamine B, phenols), hydrogen evolution reactions (HER) from water splitting, CO_2_ reduction to fuels (e.g., methanol, CO) and antibacterial photocatalytic coatings^24,25^. CuMoO_4_ can easily form heterojunctions with other semiconductors (TiO_2_, ZnO, g-C_3_N_4_, BiVO_4_, etc.), which improve band alignment for efficient charge transfer, enhance photocatalytic stability and activity, and extend the absorption range into the visible and near-infrared regions^26,27^. CuMoO₄ exists in α and γ polymorphs with different crystal structures, where α-CuMoO₄ is monoclinic and more thermodynamically stable, and γ-CuMoO₄ is triclinic, metastable, but more active photocatalytically^28^. Nanostructured CuMoO₄ (nanorods, nanosheets, etc.) increases surface area and active sites, further enhancing photocatalytic performance^19^. Given the many environmental and economic advantages of copper and molybdenum in terms of abundance, low cost, low toxicity, and chemical stability, they are suitable for scalable and sustainable photocatalytic systems^25^. Additionally, CuMoO₄ in forms II and III is synthesized under high pressure^29^. Various processes have been reviewed in the literature for the preparation of copper molybdate. For instance, Benchikhi et al. prepared α-CuMoO₄ using the sol–gel process, whereas Seevakan et al. obtained it by microwave combustion^30^. Furthermore, Wei et al. employed a solid-state method in the synthesis of α-CuMoO₄ to study the negative thermal expansion property of CuMoO₄^31^.

Regarding traditional microbial (biogenic) synthesis methods, although environmentally friendly, they often suffer from several drawbacks: poor reproducibility and limited control over particle size, shape, and crystallinity due to biological variability; impurities and organic residues from biomolecules that affect the catalytic surface activity; and slow reaction kinetics and low yield compared to hydrothermal synthesis^32^. Difficulty in scaling up for industrial production. In contrast, the hydrothermal method offers high phase purity, controlled crystal growth, tunable morphology, and higher yield in a short time. Therefore, this work demonstrates an efficient, reproducible, and scalable route to synthesize multifunctional HCM that overcomes the limitations of microbial and other conventional preparation techniques.

This research, therefore, employs the hydrothermal method to prepare HCM. Different techniques were used to evaluate the texture properties, morphology, and optical properties of the photocatalyst. Crystal violet dye was used to investigate their photoactivity under visible light. We can highlight the novelty of the current study in three main aspects: (I) the hydrothermal synthesis of HCM nanostructures that integrate both photocatalytic and antimicrobial functionalities. (II) The designed morphology, composed of interconnected rod- and plate-like units, provides many active sites for efficient degradation and microbial inhibition. Unlike earlier reports that focused either on photocatalysis or electrochemical performance, our work systematically combines dual functions within a single nanomaterial, demonstrating its practical potential for simultaneous pollutant degradation and microbial inhibition. (III) The use of response surface method (RSM) for multi-response optimization introduces a systematic approach to enhance photocatalytic efficiency. Compared with traditional microbial synthesis routes, which often suffer from poor reproducibility, slow reaction rates, and limited control over crystal structure, the hydrothermal method ensures high crystal comprehension, morphological uniformity, and scalability. Collectively, these attributes differentiate our work from traditional reports and underscore its importance as a multifunctional, cost-effective, and eco-friendly choice for wastewater treatment and microbial management.

## Experimental

### Materials

**Fig. 1:**
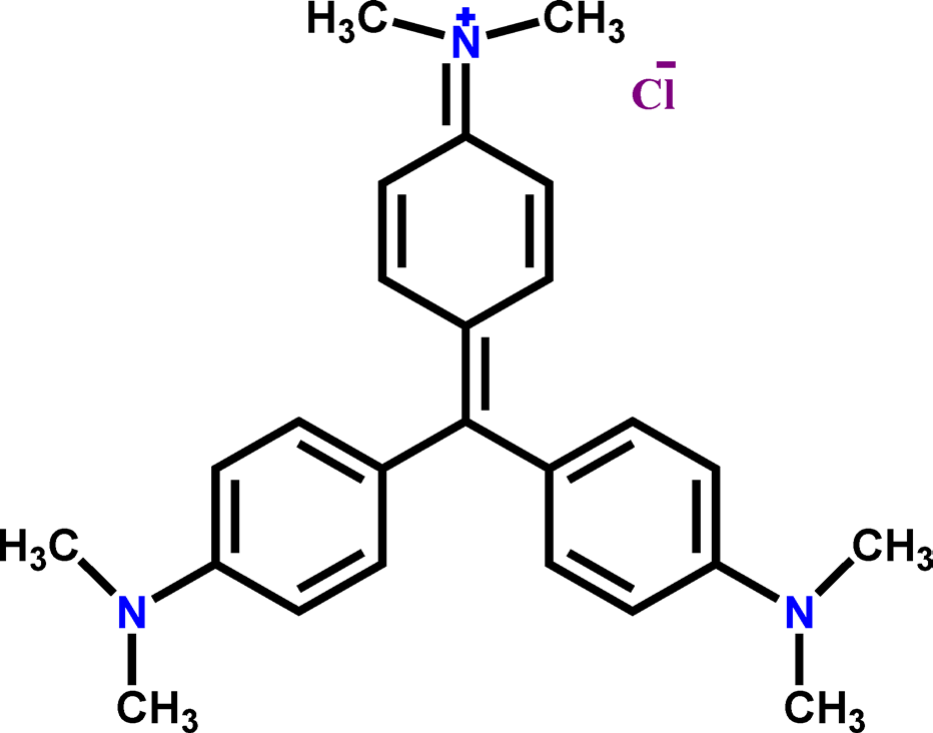
Chemical structure of crystal violet dye.

All chemicals were of analytical grade and were used without further purification. Sodium hydroxide (NaOH, ≥ 98%, pellets anhydrous), hydrochloric acid (HCl, 37%), copper acetate monohydrate (Cu (OAC)₂.H₂O, 99%), alcohol (C₂H₅OH, ≥ 95%) were purchased from Merck, Darmstadt, Germany. Crystal violet (C₂₅H₃₀ClN₃; Mo.Wt. 407.99 g/mol, (Fig. 1)) was purchased from Alpha Chemika, India. All reagents used were of analytical purity and were used as received. Deionized (DI) water was obtained from an ultrapure purifier (Ultra-pure, resistivity ≥ 18.2 MΩ).

### Synthesis of HCM

The HCM was synthesized using a modified hydrothermal method^33^. The hydrothermal synthesis route plays a crucial role in defining the crystallinity and morphology of HCM. Under high temperature and autogenous pressure, the dissolved precursors recrystallize slowly, enabling homogeneous nucleation and directional crystal growth. This results in highly crystalline, well-defined rod- and plate-like hierarchical nanostructures with reduced defect density. In contrast, conventional thermal treatments yield larger, irregular aggregates with lower crystalline due to uncontrolled grain growth and sintering. Thus, the hydrothermal environment provides both structural uniformity and morphological control that enhance the functional performance of the material.

**Fig. 2:**
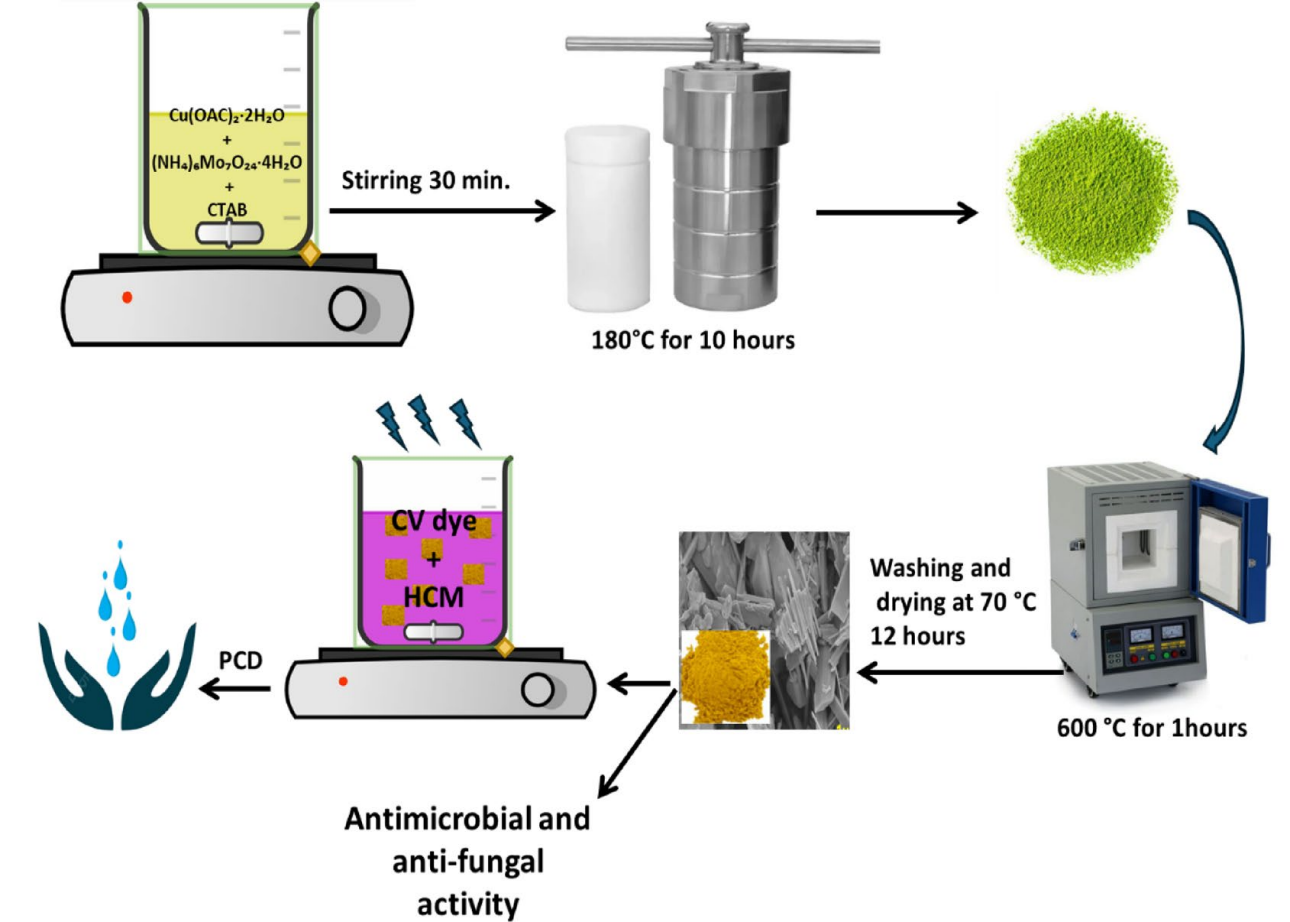
Scheme for preparation of HCM nanostructures by a hydrothermal method and their applications.

A total of 20 mmol of Cu (OAC)₂·2 H₂O and 20 mmol of (NH_4_)_6_Mo_7_O_24_·4H_2_O were dissolved in 50 mL of DI water to create a transparent solution. Subsequently, 5 mmol of cetyltrimethylammonium bromide (CTAB) was added, and then the mixture was stirred for 30 min at room temperature. The final solution was transferred into a 100 mL Teflon-lined autoclave, where it was heated to 180°C for 10 h. After the hydrothermal reaction, the sample was collected and washed using DI water. The resulting wet solid was then placed in a vacuum oven and dried at 70 °C for 12 h. Finally, the powder was calcined in a muffle furnace at 600°C for 1 h (refer to Fig. 2). The final product was washed three times using DI and was kept for further characterization and application.

### Characterization of catalysts

The X-ray diffractometer, specifically the Shimadzu XD-1 diffractometer, was used to analyze the crystallite size, characteristics, and phase description of the HCM nanostructure. The phase identification was conducted in accordance with the standards set by the Joint Committee on Powder Diffraction Standards (JCPDS). The instrumental broadening and the sample impact are the sources of peak breath in Bragg’s effect. The full widths at half maxima (FWHM) of the measured peaks were estimated and corrected according to the Pseudo-Voigt function^34^ of the line profile relation, and it can be derived as the following Eq. (1):

1 $$\beta _{{hkl}} = {\text{ }}\left[ {\beta ^{2} _{{mes.}} - ~\beta ^{2} _{{inst.}} } \right]^{{1/2}}$$

Where β_hkl_ is the corrected FWHM, β_mes_. is the measured FWHM, and β_inst_. is the instrumental FWHM.

The average Crystal Lite size (D) was determined from the broadening of the ⅩRD peaks using Scherrer’s Eq. (2)^35^.

2 $$D_{{scher}} = {\text{ }}0.9\lambda {\text{ }}/{\text{ }}\beta _{{hkl}} cos\left( {\theta _{{hkl}} } \right)$$

where *K* is a constant (0.89), *β* represents the full width at half maximum of the diffraction peak, *λ* denotes the wavelength of the X-ray radiation (measured in 0.15418 nm), and *θ* is the Bragg angle.

Lattice strain (ℇ) & placing distance (d) can be estimated using Scherrer^36^ using the following Eqs. (3, 4):

3 $$\varepsilon _{{scher}} = {\text{ }}\beta _{{hkl}} /{\text{ }}4{\text{ }}tan\left( {\theta _{{hkl}} } \right)$$

4 $$d{\text{ }} = {\text{ }}\lambda {\text{ }}/{\text{ }}2{\text{ }}sin\left( {\theta _{{hkl}} } \right)$$

The cumulative effect of crystallite size and lattice strain occurred together, but they are independent of each other and attributable to dislocation (δ), “crystal distortion and imperfections” that induce peak broadening of the as-prepared HCM nanocomposite^37^. Dislocation in a crystal may usually show defects or disorders that affect the physical and chemical properties of the crystal, so it’s important to estimate. Dislocation density (δ) was calculated according to the following Eq. (5):

5 $$\delta {\text{ }} = {\text{ }}1/D^{{2~~}} in{\text{ }}lines/{\text{ }}nm^{2}$$

The structural and chemical composition of the HCM nanostructure was characterized using a Fourier-transform infrared spectrophotometer (Nicolet Is-10 model, USA), with a vibrational frequency range from 400 to 4000 cm⁻¹, employing the KBr procedure. The morphology and surface elemental composition of the sample were analyzed using a field emission scanning electron microscope (FE-SEM; FEI Quanta FEG 250) and a high transmission electron microscope equipped with EDX (model: JEM-2100 F). The UV reflectance analysis of the prepared photocatalysts was conducted using a UV spectrophotometer (model V-570, manufactured by JASCO, Japan).

### Bench-top photocatalytic degradation (PCD) of CV dye

The performance of the prepared material in breaking down crystal violet **(CV)** dye in water was thoroughly studied. Solutions of CV were prepared by mixing a concentrated solution (250 mg/L) with deionized water to achieve the desired concentrations. In each test, a specific amount of photocatalyst was added to a volume of 10 mL of CV dye solution and stirred using a magnet to mix it well. The mixture was continuously stirred for 30 min in a closed container to allow the dye molecules to evenly attach and detach from the catalyst surface. Photocatalytic experiments were performed using a manually assembled 70 W UV lamp as the irradiation source and positioned 10 cm away from the container. Although the spectral irradiance and photon flux could not be calibrated due to the limited availability of radiometric instruments, all experiments were conducted under identical geometric and operational conditions, including fixed lamp-to-sample distance, constant ambient temperature, and continuous stirring. The results were therefore evaluated comparatively under uniform irradiation conditions. The lamp was run after a fixed warm-up time (10 min) to ensure stable emission intensity and obtain accurate results. The progress of the CV dye degradation was tracked by measuring the light absorption of the solution at 580 nm, and the amount of dye removed was calculated using the following formula for determining the degradation efficiency^38,39^ (Eq. (6)).

6 $$\:PCD\:\%=\:\frac{\left({C}_{i}-{C}_{f}\right)}{{C}_{i}}\:\times\:100\:\:\:\:$$

where C_i​_ and C_f_​ represent the initial and final CV concentrations, expressed in mg/L, respectively. The solution pH was adjusted using diluted sodium hydroxide and hydrochloric acid to assess its influence on the degradation effectiveness. After the irradiation period, the reaction mixtures were centrifuged for 10 min, and the leftover CV concentration was measured using a UV–vis spectrophotometer. Several experiments were conducted to thoroughly investigate the influence of various operational factors, including pH, irradiation time, catalyst amount, and dye concentration, on the removal efficiency of CV. Furthermore, kinetic and thermodynamic analyses were performed to have a deeper understanding of the mechanisms involved in the photocatalytic degradation of the CV dye using HCM.

### Antimicrobial activity of HCM

The antimicrobial activity of synthesized HCM nanostructure was evaluated against selected bacterial strains, including Escherichia coli, Pseudomonas aeruginosa, Bacillus subtilis, and Staphylococcus aureus. Additionally, the antifungal efficacy was assessed using Aspergillus Niger and Candida albicans. The nanoparticles were tested at concentrations of 50 and 100 micrograms per milliliter, suspended in dimethyl sulfoxide (DMSO). Chloramphenicol served as the antibacterial reference standard, while clotrimazole was used as the antifungal reference. DMSO alone was employed as a negative control. All assays were incubated at 37 degrees Celsius for 24 h. The microbial strains were obtained from the Botany and Microbiology Department, Al-Azhar University, Assiut, Egypt.

### Statistical optimization method

The Design Expert program was used to enhance batch trials for CV dye removal by employing response surface method (RSM) with a structured four-factor, three-level Box–Behnken design (BBD). This statistical design, created to provide dependable experimental systems, facilitated a thorough examination of system performance under many scenarios. This statistical approach, created to provide dependable experimental systems, facilitated an extensive examination of system performance under many settings. The model’s statistical significance was confirmed by analysis of variance (ANOVA), utilizing the p-value and Fisher’s F-test as primary assessment metrics. A p-value below 0.05, together with a high F-value, confirmed that the examined parameters significantly affected the photo-Fenton degradation process. The model’s robustness was further confirmed using the coefficient of determination (R²), with values approaching unity (R² = 1) signifying exceptional concordance between experimental and projected results. To guarantee predictive reliability, the disparity between adjusted R² and expected R² was sustained at 0.2, affirming negligible error and elevated model accuracy. To guarantee the model’s predictive accuracy, the disparity between the adjusted R² and forecasted R² was kept around 0.2, hence affirming smallest variance and substantial reliability in the forecasts. The experimental variables—pH (X1), stirring time (X2), initial CV concentration (X3), and photocatalyst dose (X4)—were manipulated at three levels (− 1, 0, and + 1), representing the minimum, median, and maximum values, as detailed in Table 1. This building facilitated a comprehensive assessment of the interactive and individual impacts of each parameter on adsorption efficiency^40^.

**Table 1:** Implicit signs and ranges in batch approach BBD experiments.

Designing parameters	Parameters	Levels		
		-1	0	+ 1
X1	pH	4	7	10
X2	Irradiation period, min.	10	35	60
X3	Initial CV concentration, mg. L^− 1^	10	15	20
X4	Dose, mg	5	10	15

Table 2 provides a detailed description of the experimental stages for each influence (X1–X4) selected for this study. The total number of experiments was 29 runs, and they were calculated using the equation (Eq. (7)) below^41^:

7 $$\:T={2}^{F}+2F+{p}_{0}$$

In this equation, T denotes the total number of experimental runs, F indicates the number of factors being analyzed, and p0 is the integer denoting the number of repetitions at the design’s central point. The factors were implicit according to the expression provided in Eq. (8)^41^.

8 $$\:{Y}_{i}=\frac{{y}_{i}-{y}_{0}}{\varDelta\:y}$$

Y_i_ stands for the value of the parameter, y_i_ is the actual value of the parameter, y_0_ is the midpoint of this parameter, and Δy is the step for all ranges of this parameter. A total of 29 laboratory runs were conducted in careful studies to investigate the effects of process conditions on performance efficiency in the CV-PCD system. The sophisticated nonlinear curve fitting method optimally applies a second-order polynomial model to the collected data, extracting the significant coefficients of this model. Below is how this quadratic prototype with linear, squared, and interaction effects among factors is calculated (Eq. (9))^42^:

9 $$\:Q={a}_{0}+\sum\:{a}_{i}y+\sum\:{a}_{ii}{y}_{i}^{2}+\sum\:\sum\:{a}_{ij}{y}_{i}{y}_{j}+\epsilon\:$$

Where Q signifies the optimal response, a₀ is the constant term, while α_i_ and α_i__i_ refer to the linear and quadratic coefficients, respectively, aij denotes the interaction coefficients among factors, and y_i_ and yⱼ denote the corresponding levels of the parameters under study. The matrix of experimental design derived from the BBD context is displayed in Table 2. To verify the repeatability and dependability of each experimental condition, it was reproduced at least three times under uniform conditions. The primary values derived from these repetitions are presented here.

**Table 2:** The experimental data for CV removal on the HCM catalyst using a 4-factor Behnken box matrix.

Run	X1	X2	X3	X4
1	7	60	20	10
2	10	35	15	15
3	7	10	20	10
4	4	35	20	10
5	4	60	15	10
6	7	60	15	15
7	7	35	20	15
8	7	60	15	5
9	7	35	15	10
10	7	35	15	10
11	7	35	20	5
12	4	10	15	10
13	7	35	10	5
14	10	60	15	10
15	4	35	15	15
16	7	35	15	10
17	7	35	15	10
18	10	10	15	10
19	7	10	10	10
20	7	10	15	15
21	7	35	10	15
22	10	35	10	10
23	10	35	20	10
24	7	10	15	5
25	4	35	10	10
26	7	35	15	10
27	7	60	10	10
28	10	35	15	5
29	4	35	15	5

## Results and discussion

### Structural characterization and optical properties of HCM nanostructure

#### ⅩRD analysis

**Fig. 3:**
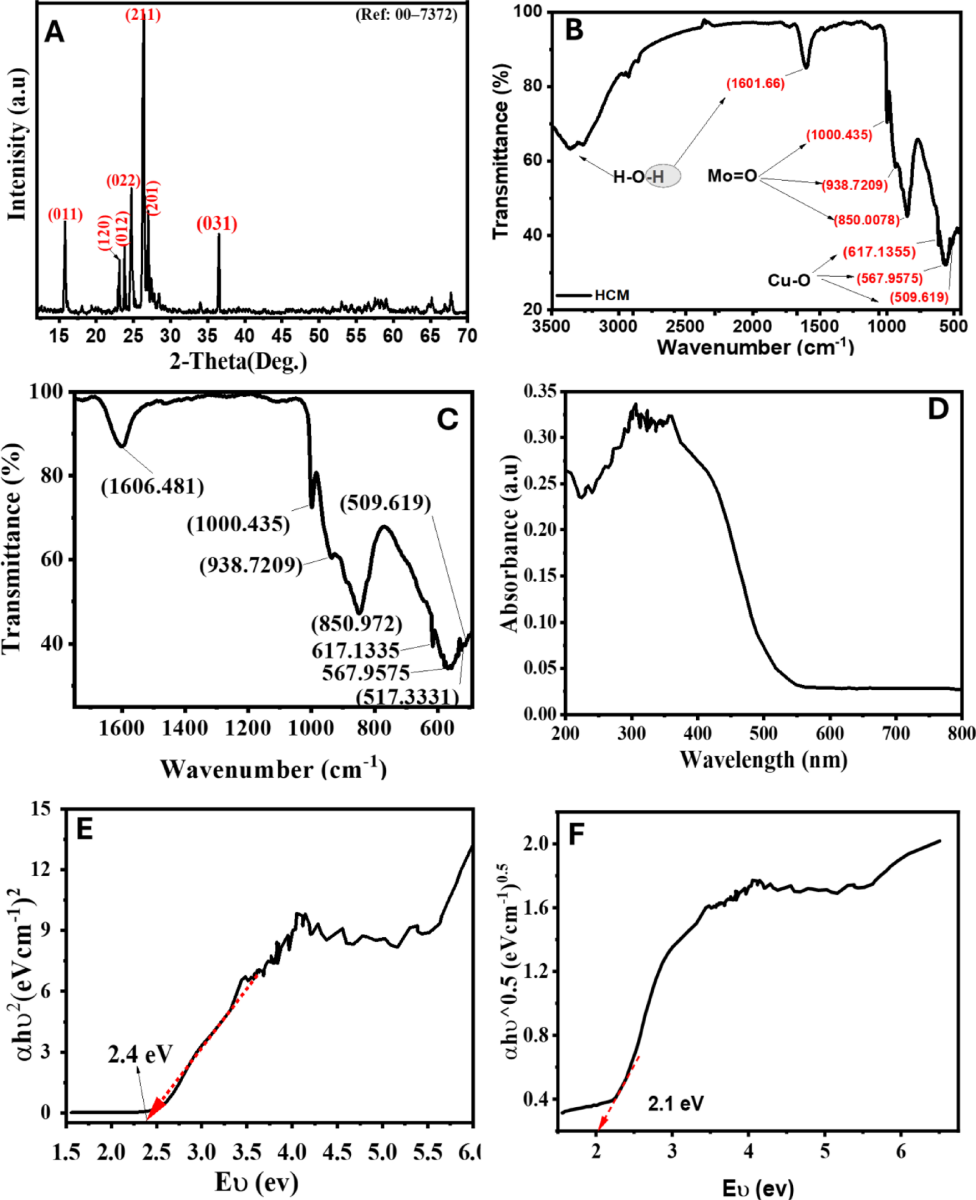
Spectra characterization of HCM: ⅩRD (**A**), FT-IR spectrum (**B** and **C**), UV-vis spectroscopy (**D**), and the direct and indirect bandgap (E and F).

Figure 3A displays the X-ray diffraction (ⅩRD) pattern of the synthesized HCM sample. The diffraction peaks are strongly defined, which confirms the material’s crystalline character. The reflections occur at 2θ of 15.81, 22.95, 23.81, 24.72, 26.42, 27.16, and 36.62º, corresponding to the crystallographic planes (011), (120), (012), (022), (211), (201), and (031), aligning well with the standard reference pattern (JCPDS card No. 00-7372)^43^. Among these, the most pronounced diffraction peak is associated with the (211) plane at around 26.6°, signifying a favored orientation along this crystallographic axis, approximately 27°, indicating a preferred orientation along this crystallographic direction. The crystallite size “Dscher” for each characteristic peak of HCM at the corresponding 2-Theta values using the Scherrer equation was tabulated as revealed in Table 3.‎.

**Table 3:** Structural XRD parameters of the as-fabricated HCM sample.

2θ˚_hkl_ Measured	Miller ‎indices			D_Scher_ nm	ε_Scher_ nm	δ lines/nm² x 10^− 3^	d _calculated_ nm	Average D_Scher_ nm
	H	k	ℓ					
15.81	0	1	1	25.95	‎0.00222	1.485	25.95	21.20
22.95	1	2	0	26.12	0.00224	1.466	26.12	
23.81	0	1	2	26.14	0.00223	1.463	26.14	
24.72	0	2	2	21.17	0.00225	2.223	21.17	
26.42	2	1	1	16.22	0.00226	3.799	16.22	
27.16	2	0	1	16.25	0.00227	3.787	16.25	
36.62	0	3	1	16.64	0.00231	3.613	16.64	

The crystallite sizes in the table range from 16.2 nm to 26.1 nm, with an average size of about 21.2 nm, indicating that the HCM sample is nanocrystalline. Smaller crystallite sizes enhance surface area, which is beneficial for photocatalytic applications. $\:{\epsilon\:}_{Scher}$ quantifies the micro strain within the crystal lattice, caused by imperfections or lattice distortions. The strain values are quite small (≈ 0.0022–0.0023), suggesting that the HCM lattice is well-ordered with minimal internal stress or defects^44^. The values range between 1.46 × 10⁻³ and 3.79 × 10⁻³ lines/nm², indicating that samples with smaller crystallite size have higher dislocation densities, consistent with the inverse relation between $\:D$ and $\:\delta\:$. A moderate dislocation density suggests a balance between crystallinity and defect concentration, which can influence photocatalytic efficiency by improving charge separation^25^. The values range between 16.22 nm and 26.14 nm (interpreted as angstroms or nanometers depending on units). These spacings correspond to the lattice planes of HCM and confirm the structural integrity and phase purity.

On the other hand, the apparent deviation between the XRD (Scherrer) and TEM particle sizes is due to the hierarchical architecture of the HCM catalyst. The TEM micrograph primarily reveals the smaller nanoparticles decorating the surface, while XRD provides an average crystallite size for the entire bulk material.

#### FT-IR spectroscopy

Fourier-transform infrared (FT-IR) spectroscopy was utilized to examine the vibrational characteristics and functional groups of the synthesized HCM sample, elucidating the bonding environment of Mo–O and Cu–O connections. Figure 3B displays the FT-IR spectrum of the prepared HCM sample, and a magnified view (inset) of the 490–1650 cm⁻¹ is shown in Fig. 3C. It exhibits unique absorption bands indicative of metal–oxygen and molybdate vibrational modes. Adsorbed water molecules (H-O-H) have a faint broad band about 1600 cm⁻¹, indicating their bending vibration. Prominent peaks in the range of 850–1000 cm⁻¹ are ascribed to the stretching vibrations of Mo = O bonds within rotational Mo^6+^ octahedra, characteristic of molybdenum oxide compounds^45^. In the lower frequency range, absorption bands identified between 500 and 600 cm⁻¹ are attributed to Cu–O stretching vibrations and lattice modes, in accordance with documented FT-IR spectra of copper molybdate phases^46^. The spectrum characteristics validate the effective synthesis of crystalline HCM, exhibiting the anticipated Cu–O and Mo–O coordination environment.

#### Optical characteristics

The UV-visible absorbance spectrum of the HCM NPs was examined by UV-Vis spectrum within the range of 200–800 nm, as shown in Fig. 3D. The spectrum exhibits a strong, broad absorption peak between 230 and 450 nm and is centered at 360 nm. The significance of the results confirmed the narrow crystalline size of the prepared product. This result was a direct consequence of the quantum confinement effect associated with nano-regime particles. The electronic band gap of semiconductors can be determined by the Tauck relationship, given as (Eqs. (10 & 11))^2,47^.

10 $$\:{\left(\alpha\:h\nu\:\right)}^{n}=A(h\nu\:-{E}_{g})$$

11 $$E_{g} = {\text{ }}1240/\lambda$$

In this equation, α is the absorption coefficient (*α* = 2.303 A/t; A is the absorbance and t in the cuvette thickness), *h* is Planck’s constant, ν is the photon frequency, and *E*_*g*_ is the optical band gap. The value of *n* could be 1/2, 3/2, 2, or 3, depending on the nature of the electronic transition responsible for absorption. The n value is 2 for a direct band gap semiconductor. According to this equation, the optical energy gap, *Eg*, of the HCM can be determined by plotting (αhѵ)² or (*αhѵ*)^⁰.⁵^ versus the photon energy *hѵ* for the direct (D) and indirect (E) bandgap HCM using the data obtained from the absorption spectra, Fig. 3 (E and F). It reveals that the obtained plotting gives a tangent to the linear portion of the curves in a certain region. The energy gap (*E*_*g*_) values are obtained by extending this straight line to intercept the (*hѵ*)-hѵ)-аxis at (αhѵ) ² = 0 or (αhѵ) ⁰.⁵ = 0. The calculated direct and indirect band gaps of HCM are 2.4 and 2.1 eV, respectively^48^. The band gap of 2.4 eV corresponds to a wavelength of approximately 517 nm, which lies within the visible-light region. Although this value suggests that HCM can absorb visible radiation, the photocatalytic experiments were performed under UV irradiation (λ = 365 nm). The photons emitted from the UV source possess energies (≈ 3.4 eV) greater than the band gap, enabling efficient excitation of charge carriers across the conduction and valence bands. The observed activity under UV light is therefore consistent with the optical characteristics of the material. Moreover, the presence of defect states and oxygen vacancies may induce sub-bandgap absorption, contributing to the extended optical response and enhanced photocatalytic efficiency. This dual sensitivity to both UV and visible light is beneficial for broad-spectrum photocatalytic applications.

### Morphology analysis (SEM, TEM, and EDX analyses)

**Fig. 4:**
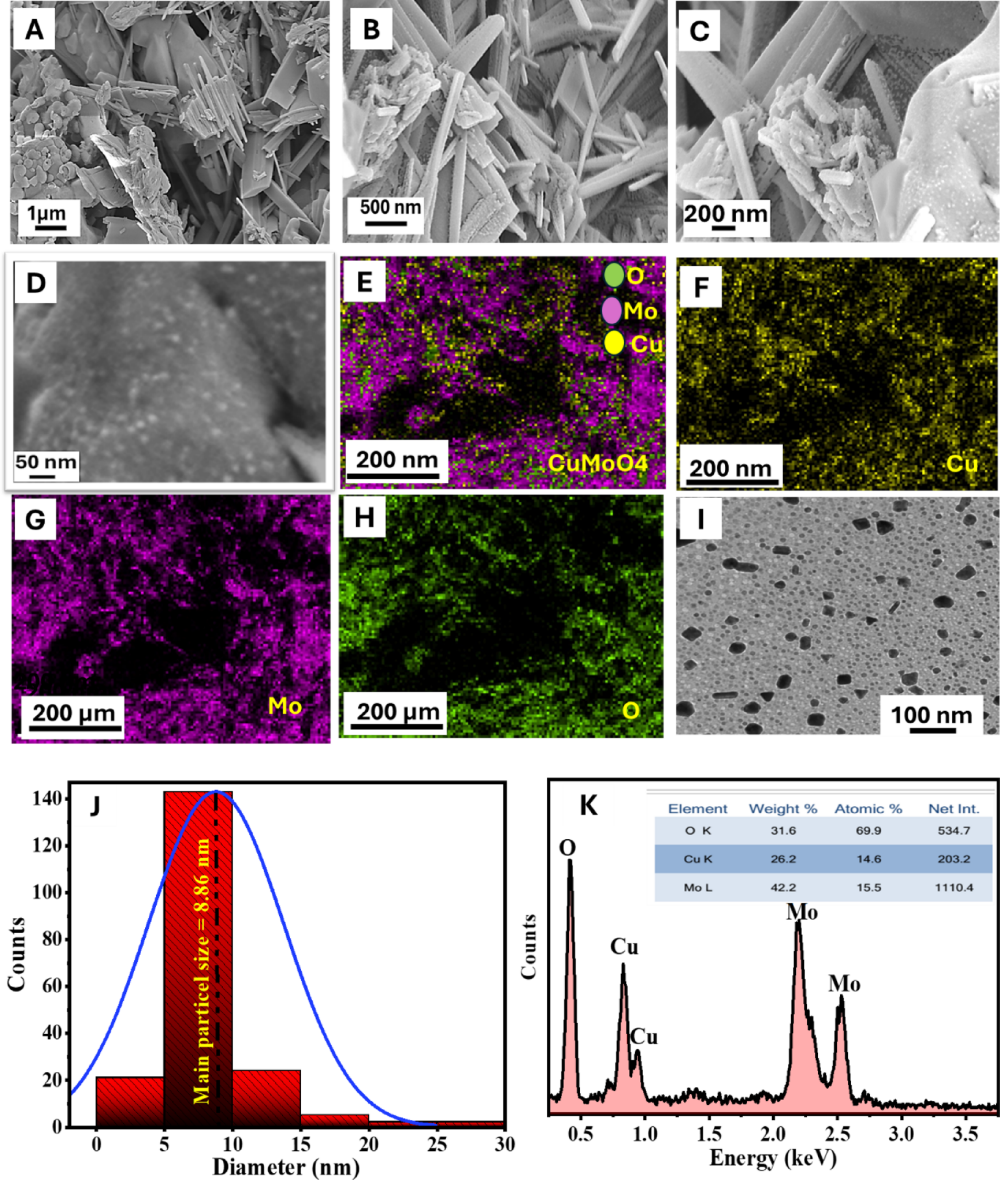
SEM images with different magnifications (**A**–**D**), elemental mapping images (**E**–**H**), TEM image (**I**), particle size distribution (**J**), and EDX spectra of HCM NPs (**K**).

The surface morphology of the produced HCM nanostructures was investigated using SEM at various magnifications (Fig. 4(A–D)). At low magnification (Fig. 4A), the sample displays tightly packed aggregates of elongated rod- and plate-like particles, signifying anisotropic crystal formation and substantial interparticle agglomeration. Nanorods range from several hundred nanometers to a few micrometers in length, with an average diameter of approximately 100–200 nm. A higher magnification view (Fig. 4B) displays well-defined nanorods with sharp edges and relatively smooth surfaces, which are coupled to form a porous network. At higher magnifications, Fig. 4C shows a hierarchical morphology of rod-like structures (100–300 nm in diameter) adorned with smaller irregular nanoparticles (~ 20–50 nm), resulting in increased surface roughness and surface-to-volume ratio. At the maximum magnification (Fig. 4D), ultrafine nanoparticles (< 20 nm) are clearly detected on the surface of the nanorods, indicating the presence of many active sites and a diverse surface. This hierarchical arrangement of rod- and plate-like structures with nanoscale ornamentation is expected to improve the material’s functional capabilities, especially in catalytic and electrochemical applications.

#### EDX mapping

Figures 4 (E-H) show the energy-dispersive X-ray spectroscopy (EDX) elemental mapping of the produced HCM nanostructure, showing its uniform distribution of constituent elements. The combined mapping image (Fig. 4E) reveals the presence of copper (Cu), molybdenum (Mo), and oxygen (O), indicating successful creation of the CuMoO_4_ phase. The elemental map of Cu (Fig. 4F) shows homogeneous dispersion across the surface, whereas Mo (Fig. 4G) looks evenly dispersed with no noticeable clumping. Similarly, oxygen mapping (Fig. 4H) shows a consistent distribution that corresponds well to the expected oxide framework. Co-localization of Cu, Mo, and O signals validates the stoichiometric composition and purity of the produced HCM, consistent with prior studies on transition metal oxides, especially copper and molybdenum oxides^48,49^. These data show that the EDX mapping validates the structural integrity and successful synthesis of the targeted molecule. The EDX results obtained clearly confirm the ⅩRD data, which indicate the formation of HCM in its pure form.

#### EDS spectroscopy

Figure 4K illustrates an energy-dispersive X-ray spectroscopy (EDS) spectrum, providing elemental analysis of the examined sample. The spectrum displays specific peaks associated with oxygen (O) at roughly 0.41 keV^47,50^. Two peaks appeared at approximately 0.82 and 0.93 keV, which are attributed to the element copper (Cu)^51^. Two peaks were also observed at 2.19 and 2.5 keV, which are attributed to the element molybdenum (Mo)^52^. The O peak signifies oxide production or oxygen incorporation in the sample, whilst the Cu signals imply either substrate contribution or sample makeup that includes copper. The pronounced and acute Mo peaks validate the existence of molybdenum as a principal element in the material.

#### TEM analysis

Figure 4I exhibits an image of the prepared HCM obtained using a transmission electron microscope (the fraction containing the smallest particles was selected from the prepared sample), which reveals their approximately spherical shape and relatively uniform distribution on the surface. Additionally, the image demonstrates that the particles are uniformly disseminated with minimal aggregation, signifying successful synthesis and stability. The nanoparticle size distribution is further examined in Fig. 4J, which displays a histogram fitted with a Gaussian curve^53,54^. The research reveals that most particles are within the nanoscale region, with an average particle size of approximately 19.86 nm. A small size distribution confirms a large surface area and leads to a larger active surface, which is a key element influencing the performance of the prepared material.

####  BET analysis

**Fig. 5:**
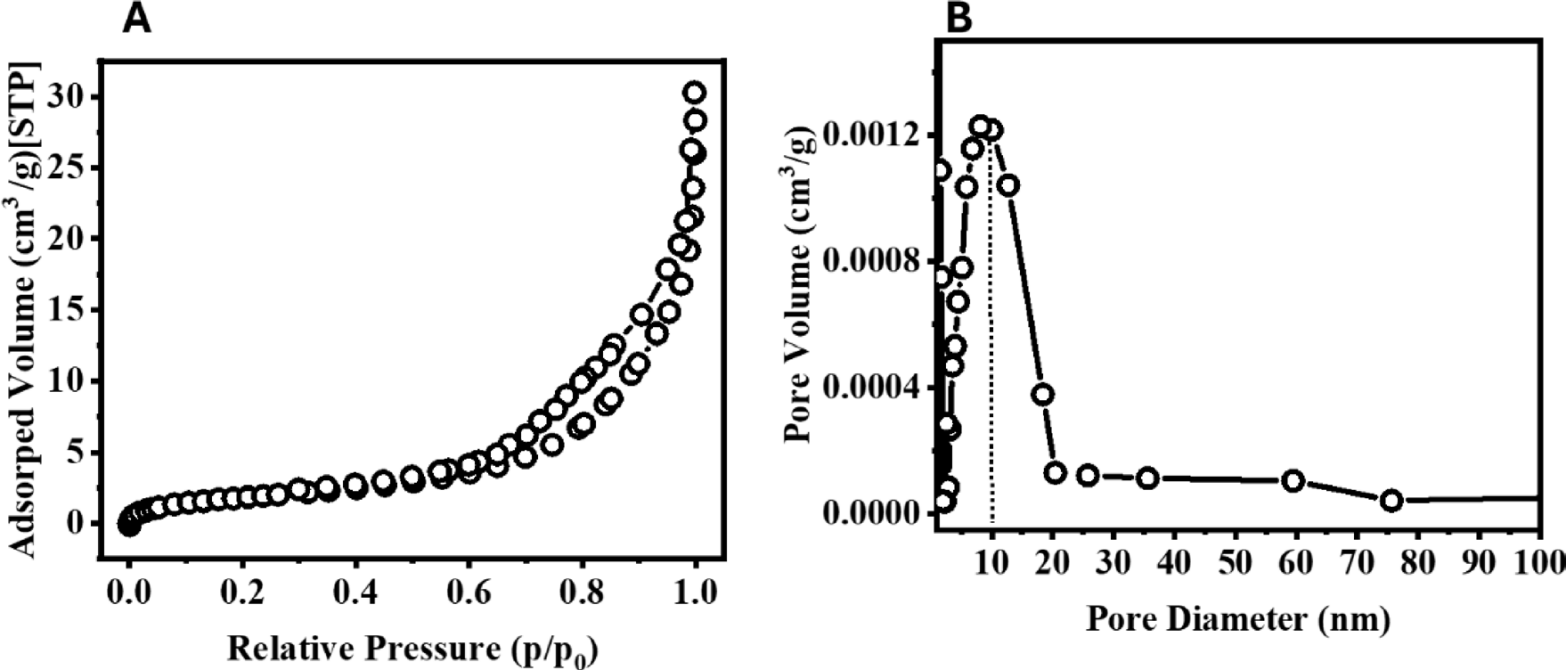
N₂ adsorption/desorption and pore size distribution for the synthesized HCM.

The N_2_ adsorption/desorption analysis of the synthesized HCM (Fig. 5A) displays a characteristic type IV isotherm with an H_3_ hysteresis loop, indicating the presence of mesoporous structures formed by the aggregation of nanosized particles. Moreover, the corresponding BJH pore size distribution (Fig. 5b) shows a dominant pore diameter centered around 12.35 nm, which is consistent with mesoporous characteristics. The BET surface area was determined to be 37.65 m²/g, suggesting a large accessible surface area that enhances dye molecule adsorption and photocatalytic efficiency. The coexistence of mesopores and hierarchical morphology promotes efficient mass transport and active-site accessibility during photocatalytic degradation.

### Design and optimization of experiments for dye adsorption

**Fig. 6:**
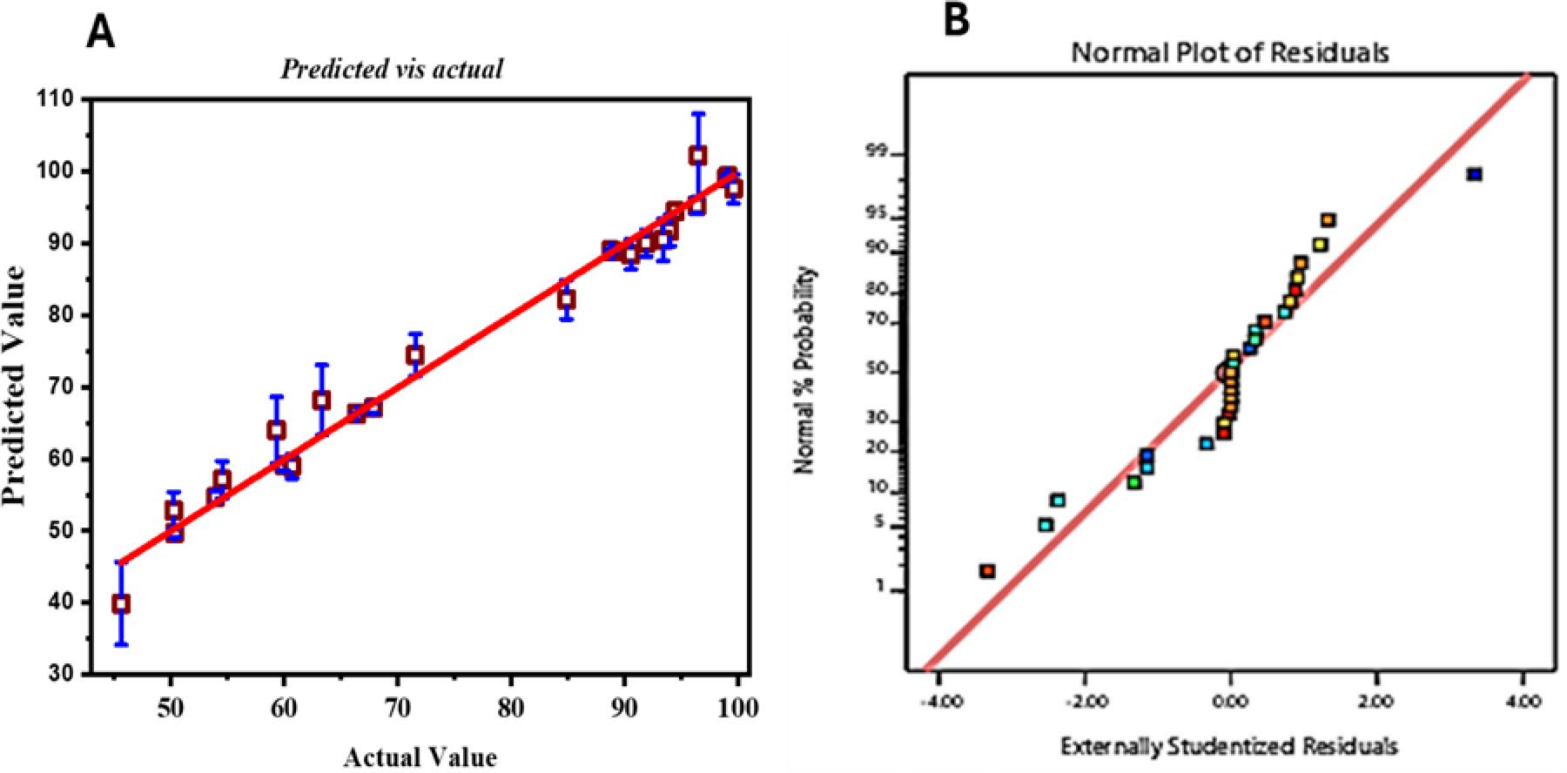
Regression analysis diagnostic plots: The correctness of the model is demonstrated by the predicted versus actual values with bar error (**A**); a normal probability plot of the residuals demonstrates normalcy (**B**).

Figure 6 (A and B) depict the investigative assessment of the regression typically employed to investigate the photocatalytic degradation of crystal violet. Figure 6A presents the parity plot comparing predicted and experimental degradation efficiencies. The close distribution of data points along the 45° line with a high R² (0.992) indicates the reliability of the quadratic RSM model. Error bars represent the standard deviation of triplicate runs, confirming satisfactory reproducibility. The optimized parameters, pH 10, irradiation time of 60 min, and 15 mg of catalyst dosage, have direct physical relevance to the photocatalytic mechanism. The alkaline medium promotes dye adsorption and hydroxyl-radical formation, prolonged irradiation enhances charge-carrier separation, and suitable catalyst loading ensures sufficient active sites without excessive light shielding. These interactions explain the experimentally observed maximum CV-removal efficiency under the optimized conditions. Figure 6B shows the normal probability plot of the externally studentized residuals, which was used to check if the regression model assumes normality. The residuals are closely aligned with the reference line, suggesting that the error distribution is approximately normal. This supports the use of regression analysis. Small deviations at the ends of the plot might indicate possible outliers, which could result from variations in the adsorption process under specific experimental conditions. Overall, these results confirm that the regression model is a dependable method for describing the adsorption behavior of CV, with minor errors that have little effect on the overall conclusions.

Table 4 displays ANOVA results for the simplified quadratic model used to analyze the PCD of CV dye by the HCM catalyst. The model was statistically significant (F = 55.01, *p* < 0.0001), and its fit indicated that it is appropriate to use to explain the observed variance in CV removal. From the factors studied, pH (A) (F = 204.77, *p* < 0.0001), irradiation time (B) (F = 18.46, *p* < 0.0001), and amount of catalyst dosage (F = 41.90, *p* < 0.0001) were found to be the more substrate-dependent variables influencing for the removal process and its efficiency. These results emphasize that operational conditions, especially pH, reaction time, and the amount of catalyst used, are key factors influencing the efficiency of decolorizing the CV dye. The quadratic terms A^2^ and B^2^ (F = 162.90, *p* < 0.0001) and (F = 87.42, *p* < 0.0001), respectively, highlight the nonlinear dependence of CV removal on pH value and reaction time. On the other hand, the interaction between C-Conc. and D-Dose (CD, F = 10.76, *p* = 0.0055) reveals a substantial combined influence of both parameters on CV removal efficiency. The residual sum of squares (172.08) was comparatively low, indicating that the model left only minimal variability unexplained. The nonsignificant lack of fit (*p* = 0.11) confirms that the model adequately describes the experimental data, indicating an acceptable agreement between the predicted and observed values.

The regression coefficients, confidence limits, and variance inflation factors (VIF) for the quadratic model describing CV degradation are reported in Table 5. The value of the intercept, 94.45, represents high baseline efficiency for CV removal. Among the linear parameters, pH (A, coefficient = 14.48, CI: 12.31–16.65), irradiation time (B, coefficient = 16.69, CI: 14.52–18.86), and catalyst dosage (D, coefficient = 6.55; 95% CI: 4.38–8.72) were found to significantly enhance CV degradation, while dye concentration (C, coefficient = 1.45, 95% CI: − 0.72–3.62) showed little influence. Interaction effects were also significant, as seen for effect AB (coefficient = 7.04, CI: 3.29–10.80), confirming the interaction pattern qualitatively described in Table 5. This was further established by quadratic terms A^2^ (coefficient: -17.57, CI: -20.52 to -14.62) and B^2^ (coefficient = -12.87, CI: -15.82 to -9.92), which reconfirmed the non-linear effect of pH and reaction time on CV removal explicitly. The fact that all VIFs were close to one means that there was no multicollinearity among the model terms. From these results, we collectively emphasize irradiation time and pH and their possible interaction as main effects controlling the efficiency of CV dye degradation, with the statistical significance of both linear and quadratic terms indicating the adequacy of the proposed quadratic model in the optimization of the photocatalytic process^55^.

**Table 4:** Results of the ANOVA for CV dye degradation based on the simplified quadratic model using bimetallic HCM.

Source	Sum of squares	df	Mean square	F-value	*p*-value	
Model	9466.63	14	676.19	55.01	< 0.0001	Significant
A-pH	2516.91	1	2516.91	204.77	< 0.0001	
B-Time	3342.01	1	3342.01	271.90	< 0.0001	
C-Conc.	25.23	1	25.23	2.05	0.1739	
D-Dos.	514.96	1	514.96	41.90	< 0.0001	
AB	198.53	1	198.53	16.15	0.0013	
AC	15.64	1	15.64	1.27	0.2782	
AD	12.04	1	12.04	0.9796	0.3391	
BC	27.20	1	27.20	2.21	0.1591	
BD	5.04	1	5.04	0.4100	0.5323	
CD	132.25	1	132.25	10.76	0.0055	
A²	2002.22	1	2002.22	162.90	< 0.0001	
B²	1074.47	1	1074.47	87.42	< 0.0001	
C²	22.39	1	22.39	1.82	0.1985	
D²	124.53	1	124.53	10.13	0.0066	
Residual	172.08	14	12.29			
Lack of Fit	172.08	10	17.21	29.96	0.11	Non-significant
Pure error	0.0000	4	0.0000			
Cor total	9638.71	28				

**Table 5:** Result of the ANOVA for the quadratic model coefficients for CV dye elimination by bimetallic HCM.

Factor	Coefficient estimate	df	Standard Error	95% CI Low	95% CI High	VIF
Intercept	94.45	1	1.57	91.09	97.81	
A-pH	14.48	1	1.01	12.31	16.65	1.0000
B-Time	16.69	1	1.01	14.52	18.86	1.0000
C-Conc.	1.45	1	1.01	-0.7207	3.62	1.0000
D-Dos.	6.55	1	1.01	4.38	8.72	1.0000
AB	7.04	1	1.75	3.29	10.80	1.0000
AC	− 1.98	1	1.75	− 5.74	1.78	1.0000
AD	− 1.73	1	1.75	− 5.49	2.02	1.0000
BC	− 2.61	1	1.75	− 6.37	1.15	1.0000
BD	− 1.12	1	1.75	− 4.88	2.64	1.0000
CD	− 5.75	1	1.75	− 9.51	− 1.99	1.0000
A²	− 17.57	1	1.38	− 20.52	− 14.62	1.08
B²	− 12.87	1	1.38	− 15.82	− 9.92	1.08
C²	− 1.86	1	1.38	− 4.81	1.09	1.08
D²	− 4.38	1	1.38	− 7.33	− 1.43	1.08

The final regression equation was derived from the coefficients listed in Table 5, which reflect the relative contribution of each parameter as well as their interaction effects. Accordingly, the quadratic model can be expressed as follows (Eq. (12)):

12 $$\begin{gathered} PCD{\text{ }}efficiency{\text{ }}\left( \% \right){\text{ }} = {\text{ }}94.45{\text{ }} + {\text{ }}14.48A{\text{ }} + {\text{ }}16.69B{\text{ }} + {\text{ }}1.45C{\text{ }} + {\text{ }}6.55D{\text{ }} + {\text{ }}7.04AB{\text{ }} \hfill \\ - {\text{ }}1.98AD{\text{ }} - {\text{ }}1.73BC{\text{ }}{-}{\text{ }}2.61BD{\text{ }} - {\text{ }}1.575A{\text{ }} - {\text{ }}17.57B{\text{ }} - {\text{ }}12.87C{\text{ }} - {\text{ }}1.86{\text{ }} - {\text{ }}4.38D \hfill \\ \end{gathered}$$

The statistically significant coefficients have made it clear that pH (A), irradiation time (B), and catalyst dosage (D) are the primary affecting factors of CV degradation. The positive coefficients for pH, time, and dosage, 14.48, 16.69, and 6.55, respectively, clearly state their strong roles in enhancing CV removal efficiency of the designed catalyst. The low coefficient for dye concentration, only as much as 1.45, expresses negligible influence; perhaps there is insufficient competition for active photocatalytic sites. A positive interaction term between pH and reaction time, indicated by AB, is 7.04, while a negative quadratic coefficient for time, B^2^, reveals efficiency at higher irradiation periods. From the main effects, interaction terms, and quadratic contributions that can be combined into forming a comprehensive model structure, toward searching for an optimal set of operating parameters to maximize the PCD efficiency. By integrating the main effect, interaction terms, and quadratic contribution, the model provides a comprehensive framework for optimizing operating parameters to maximize PCD efficiency. Such insights are valuable for improving the performance of photocatalyst in environmental remediation practice.

### Optimization and response surface methodology

**Fig. 7:**
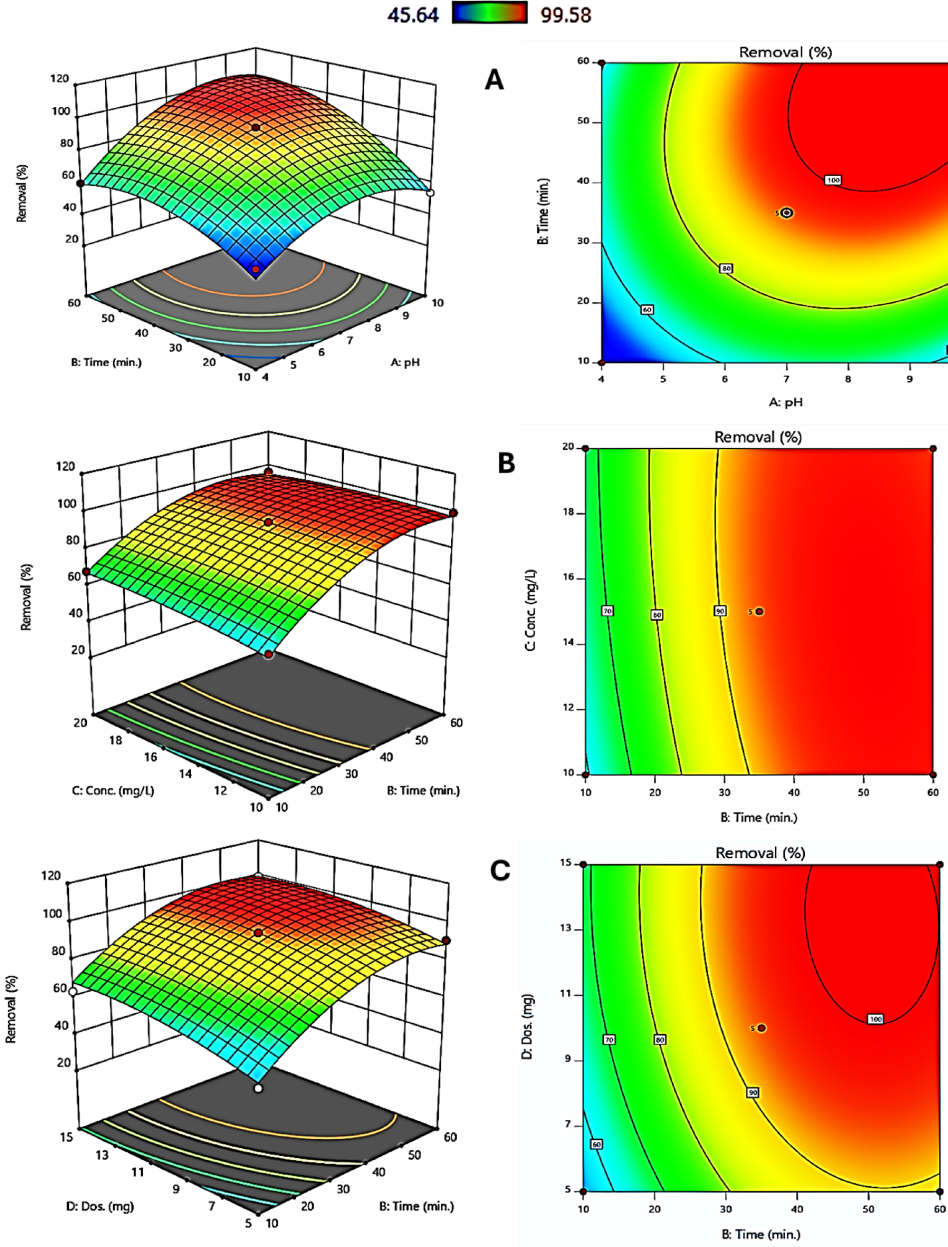
The 2-D contour plots and 3D-response surface plots (**A**, **B**, and **C**) show how essential parameters interact to determine CV dye PCD efficiency. The figures show how pH, reaction duration, catalyst dose, and dye concentration affect the degradation process.

Three-dimensional response surface plots, together with two-dimensional contour maps, were used in describing the influence of major operating parameters on the efficiency of photocatalytic degradation (PCD) of CV dye by synthesized HCM catalyst. These visualizations clearly bring out how pH and irradiation time, catalyst dosage, and dye concentration interact to manifest a combined effect on the overall degradation performance. Figure 7A depicts the relationship between pH and reaction time in terms of CV removal efficiency. The response surface reveals that longer reaction periods result in much higher removal efficiency, particularly from mildly to alkaline pH levels. Supreme efficiency is achieved at a pH value of 10 and an irradiation duration of 60 min. Degradation efficiency decreases under moderate acidic pH values lower than 7 or high alkaline medium pH values higher than 10 conditions, indicating insufficient production of reactive oxygen species under such conditions. These results show that the HCM photocatalyst functions best at moderate alkaline pH levels, providing an important critical parameter for efficient degradation.

Figure 7B exhibits the interaction between CV dye initial concentration and reaction time. The response surface exposes a positive association value between both parameters and PCD efficiency, with the highest efficiency observed at CV dye concentrations of 15 mg/L or less and irradiation periods of 60 min. Moreover, the response surface indicates that increased dye concentrations adversely impact removal efficiency, even with extended reaction times. The contour map corroborates this tendency, depicting a shift from reduced efficiency at higher CV concentrations and brief reaction durations to optimal efficiency at lower CV dye concentrations and prolonged reaction times. These results emphasize the importance of not exceeding a certain concentration limit to match the active sites on the catalyst surface. Additionally, this behavior is most likely owing to competition for active sites on the HCM photocatalyst surface and decreased light penetration induced by increasing solution opacity at higher CV dye concentrations.

Figure 7C illustrates the investigation of the connection between reaction time and catalyst dosage. The response surface indicates that an increased catalyst dosage enhances removal efficiency, even with brief reaction durations. Optimal degradation occurs with a catalyst dose less than or equal to 10 mg and a shorter reaction time less than or equal to equal to 60 min, while a catalyst amount below 10 mg results in reduced degradation efficiency, as illustrated by the blue and green areas in the contour plot. These findings underscore the necessity of ensuring sufficient catalyst availability to sustain optimal photocatalytic activity and sufficient active sites. The surface response on the contour plots offers a comprehensive evaluation of the interactions among key parameters governing the removal of CV dye. The results refer to the optimal CV degradation effectiveness attainable by functioning at a moderate alkaline pH (pH 10), employing elevated catalyst doses, extending reaction durations, and utilizing reduced initial concentrations of CV dye. The findings agree with the ANOVA results, which demonstrated that irradiation time, catalyst dosage, and pH, together with their interactions, exert significant effects on the degradation process. Furthermore, these outcomes offer practical guidance for improving photocatalytic systems and advancing their use in environmental remediation.

### Influence of individual factors

**Fig. 8:**
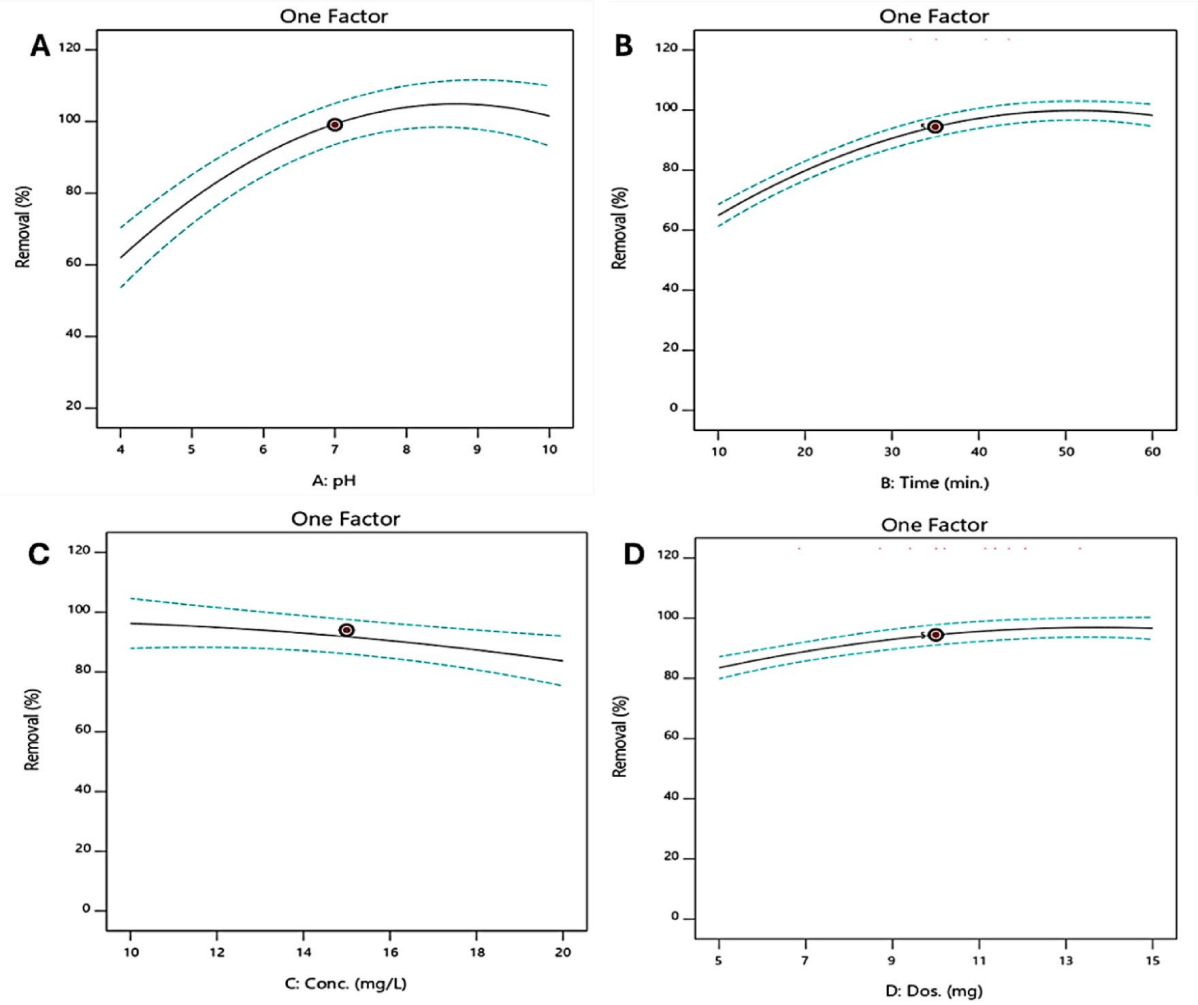
The plots of the independent impacts of (**A**) pH, (**B**) irradiation time, (**C**) CV initial concentration, and (**D**) HCM dosage on the removal efficiency of CV dye.

Figure 8 shows the independent effects of pH, irradiation time, catalyst dose, and dye concentration on the photocatalytic degradation (PCD) efficiency of the CV dye. Single-factor response plots illustrate the effect of each parameter on dye removal under controlled conditions. As shown in Fig. 8A, degradation efficiency increases with increasing pH, reaching a maximum under moderately basic conditions (around pH 10), confirming the strong dependence of the photocatalyst-dye interaction on solution acidity. In Fig. 8B, the removal efficiency improves gradually with reaction time and stabilizes after approximately 60 min, suggesting that equilibrium is reached after sufficient UV exposure and catalyst-dye interaction. The plot in Fig. 8C shows a decrease in efficiency with increasing dye concentration, which can be attributed to stronger competition between dye molecules for the limited active sites on the HCM surface and a reduction in the number of reactive species at higher concentrations. Finally, Fig. 8D shows that increasing the catalyst dose accelerates dye degradation in an almost linear manner; however, this improvement becomes less pronounced at higher loadings, possibly due to catalyst agglomeration or saturation of the active sites. Taken together, these observations highlight the critical need to optimize operational parameters to maximize degradation efficiency and facilitate the practical application of HCM photocatalysts in environmental remediation.

### Variable optimization through desirable operations

Variable optimization has always been considered an important phase of mathematical modeling and experiments. It seeks to identify the values of independent variables that will elicit the most favorable response from the system. The Derringer desirability function (DDF), originally proposed by Derringer and Suich in 1980, is one of the most widely used multi-response optimization approaches. Since responses are many in most real processes, the desirability function approach was developed to overcome this problem. Each response is transformed into a standardized desirability value, which ranges from 0 to 1, with 1 representing the most desirable outcome and 0 representing the least desirable result. A combination of individual desirability functions into a global desirability function allows finding optimal operating conditions for maximization, minimization, or even targeting a particular value that satisfies several objectives simultaneously. The comprehensive desirability score is computed using the following mathematical formula (Eq. 13)^56,57^.

13 $$\:D=\left({d}_{1}\times\:{d}_{2}\times\:\dots\:\dots\:\:\times\:{d}_{n}\right)\frac{1}{n}=({\prod\:}_{i=1}^{n}{d}_{1})\frac{1}{n}$$

In this situation, D symbolizes the general desirability, n denotes the full number of variables considered, and d_i_ refers to the independent desirability of each response. The primary goal of the DDF approach is to determine the operating conditions that maximize the general desirability value. The method used in the study provides a robust and well-established framework for simultaneously optimizing multiple parameters, namely, pH symbolized by A, irradiation time symbolized by B, initial CV dye concentration symbolized by C, and catalyst dosage symbolized by D, to achieve the highest possible photocatalytic CV degradation efficiency.

**Fig. 9:**
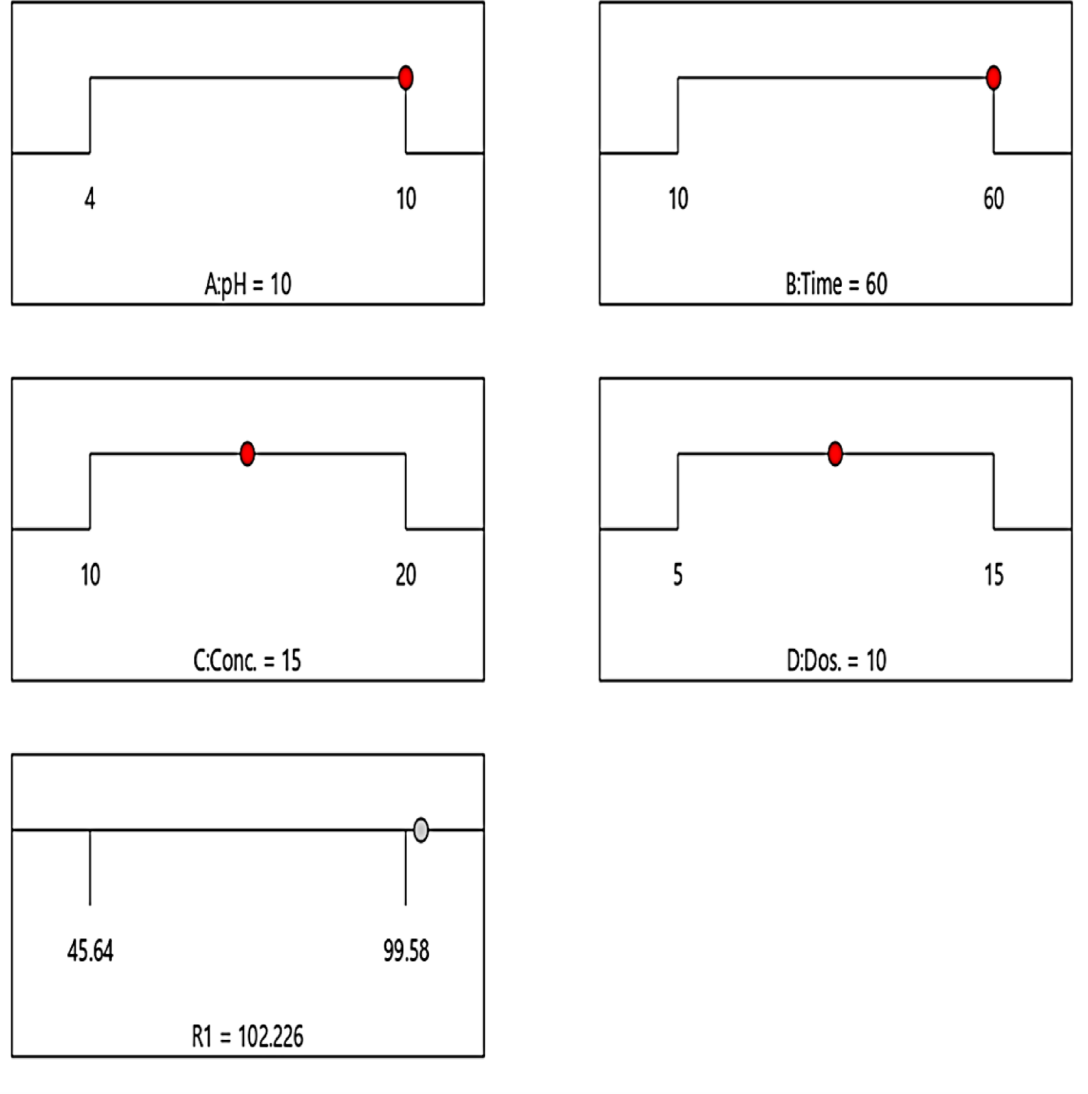
The optimized conditions for CV dye removal: pH (10), irradiation duration (60 min), CV dye concentration (15 mg/L), and catalyst dose (15 mg), resulting in 100.85% elimination efficiency and a desirability of 1.0.

Figure 9 presents the optimization data obtained for the photocatalytic degradation of the crystal violet using the HCM photocatalyst. Optimum values ​​for key operating parameters—pH, irradiation periods, catalyst dose, and initial dye concentration—were studied extensively. The optimization process was guided by a quadratic regression model, which found the best solution by achieving a maximum PCD yield with a goal value of 1.0. The optimal pH was set at 10, at which the HCM catalyst exhibited the highest activity. The response time was refined to 60 min, indicating that prolonged irradiation is necessary for efficient deterioration. The best yield was achieved with a catalyst dosage of 20 mg, which ensured sufficient availability of active sites while maintaining resource efficiency. The optimum initial dye concentration was determined to be 15 mg/L, indicating that lower concentrations promote degradation due to less competition for active sites and greater light penetration. The predicted removal efficiency was achieved at 100.05% under these ideal conditions, which means that CV was almost completely broken down. The desirability score of 1.0 validates both the reliability of the regression model and the success of the optimization strategy. These results emphasize the critical role of process optimization in improving photocatalytic performance and provide a practical framework for applying HCM photocatalysts in environmental applications.

To further evaluate the efficiency of the as-synthesized HCM photocatalyst, its performance in the degradation of crystal violet was compared with that of similar photocatalysts reported in the literature. Table 5 summarizes key experimental parameters and removal efficiencies. The data reveal that the HCM prepared in this study achieved nearly complete degradation (≈ 99%) within 60 min, outperforming or matching many reported metal molybdate and oxide catalysts. The enhanced activity is attributed to its hierarchical nanostructure and synergistic Cu–Mo redox behavior that is detailed below, which facilitates charge transfer and reactive oxygen species generation.

On the other hand, the current work focused on the function of the unique morphological structure of the synthesized HCM nanostructure and studied its efficiency in photocatalytic degradation and biological activity. TiO_2_ and other conventional photocatalysts were not employed, as the primary goal was to examine the intrinsic photocatalytic behavior of the synthesized HCM nanostructure. Although degradation experiments were carried out under UV illumination to maintain reproducibility, the narrower band gap of HCM (~ 2.4 eV) suggests potential activity under visible light. Moreover, the presence of Cu and Mo ions redox pairs enhances charge separation efficiency, distinguishing HCM from traditional wide-band-gap catalysts such as TiO_2_. Moreover, Table 6 exhibits the comparison between the synthesized HCM nanostructure, the TiO_2_ photocatalyst, and other materials that have been used in the photocatalytic degradation of CV dye.

**Table 6:** Comparing PCD efficiency using different reported materials for CV degradation.

Catalyst	Efficiency of dye removal (%)	Optimum conditions	Refs.
TiO_2_/clinoptilolite composites	98	pH = 6; the concentration of CV dye is 15 mg/L; irradiation time is 180 min; and catalyst dose is 50 mg at room temperature.	^58^
Nanochitosan/carboxymethylcellulose/TiO_2_biocomposite	95	pH = 6; the concentration of CV dye is 200 mg/L; irradiation time is 180 min; the catalyst dose is 200 mg; at room temperature.	^59^
TiO₂/CP	88.5	pH range 5.5–6.5 CV; the concentration of CV dye is 10 mg/L; the catalyst dose is 1 M TiO₂/CP; the irradiation time is 120 min at room temperature.	^60^
Silver vanadium oxides	97	pH = 7, the concentration of CV dye is 10 mg/L, and the irradiation time is 24 h at room temperature.	^61^
copper and silver co-doped nanocrystalline MgFe_2_O_4_	93	pH 9; the concentration of CV dye is 10 mg/L, the irradiation time is 120 min, and the catalyst dose is 20 mg at room temperature.	^62^
Pd@NC nanocomposite	~ 95.5	pH = 10; the concentration of CV dye is 10 mg/L; 20 mg of sorbent per 20 mL of dye, and the irradiation time is 60 min and at room temperature.	^63^
Nickel ferrite nanoparticles	95	pH = 11; the concentration of CV dye is 10 mg/L, the catalyst dose is 25 mg per 50 ml of dye, and the irradiation time is 60 min at room temperature	^64^
Xanthated rice husk (XRH) rice husk	96.16	pH = 10; the concentration of CV dye is 50 mg/L, the catalyst dose is 25 mg per 25 ml of dye, and the irradiation time is 70 min at room temperature.	^65^
Hierarchical CuMoO_4_ (HCM) nanostructure	< 99	pH = 10; the concentration of CV dye is 15 mg/L, the catalyst dose is 20 mg per 10 ml of dye, and the irradiation time is 60 min at room temperature.	This work

### Kinetics of CV removal on HCM

The PCD of crystal violet (CV) dye using HCM photocatalyst was investigated through a kinetic study to understand the reaction mechanism and efficiency. A concentration of 0.15 mg/L of CV dyes was irradiated in the presence of 10 mg of HCM photocatalyst at pH = 10 at different times, then the remaining concentration was measured carefully spectrophotometrically. Kinetic data was analyzed using two models, pseudo-first and second-order models. The reaction was observed during a period of 1 to 60 min under UV light exposure. The pseudo-first order (PFO) model and its half-life period ($\:{t}_{0.5})\:$were expressed, respectively, according to the equations below (Eqs. (14 & 15))^63^.

14 $$\:\mathrm{l}\mathrm{n}\left(\raisebox{1ex}{${C}_{o}$}\!\left/\:\!\raisebox{-1ex}{${C}_{t}$}\right.\right)={K}_{1}\:\times\:\:t\:\:\:\:\:\:\:$$

15 $$\:{t}_{0.5}=0.693/{K}_{1}$$

C_o_ denotes the initial concentration of CV dye, whereas C_t_ signifies the residual CV concentration after a particular period, and K_1_ refers to the rate constant of the PFO model. Also, the pseudo-second order (PSO) model and its half-life period were given by the following equations, respectively (Eqs. (16 & 17))^66^.

16 $$\:\frac{1}{{C}_{t}}=\:{\frac{1}{{C}_{o}}+K}_{2}\times\:t\:\:\:\:$$

17 $$\:{t}_{0.5}=\raisebox{1ex}{$1$}\!\left/\:\!\raisebox{-1ex}{${K}_{2}{C}_{0}$}\right.$$

Where K_2_ represented the rate constant of the PSO model, the kinetic behavior of CV degradation over the HCM photocatalyst was analyzed using the pseudo-first-order model, which is widely employed in photocatalysis. Although other kinetic models—such as the pseudo-second-order and Elovich equations—are often applied in adsorption or coupled systems^67,68^.

**Fig. 10:**
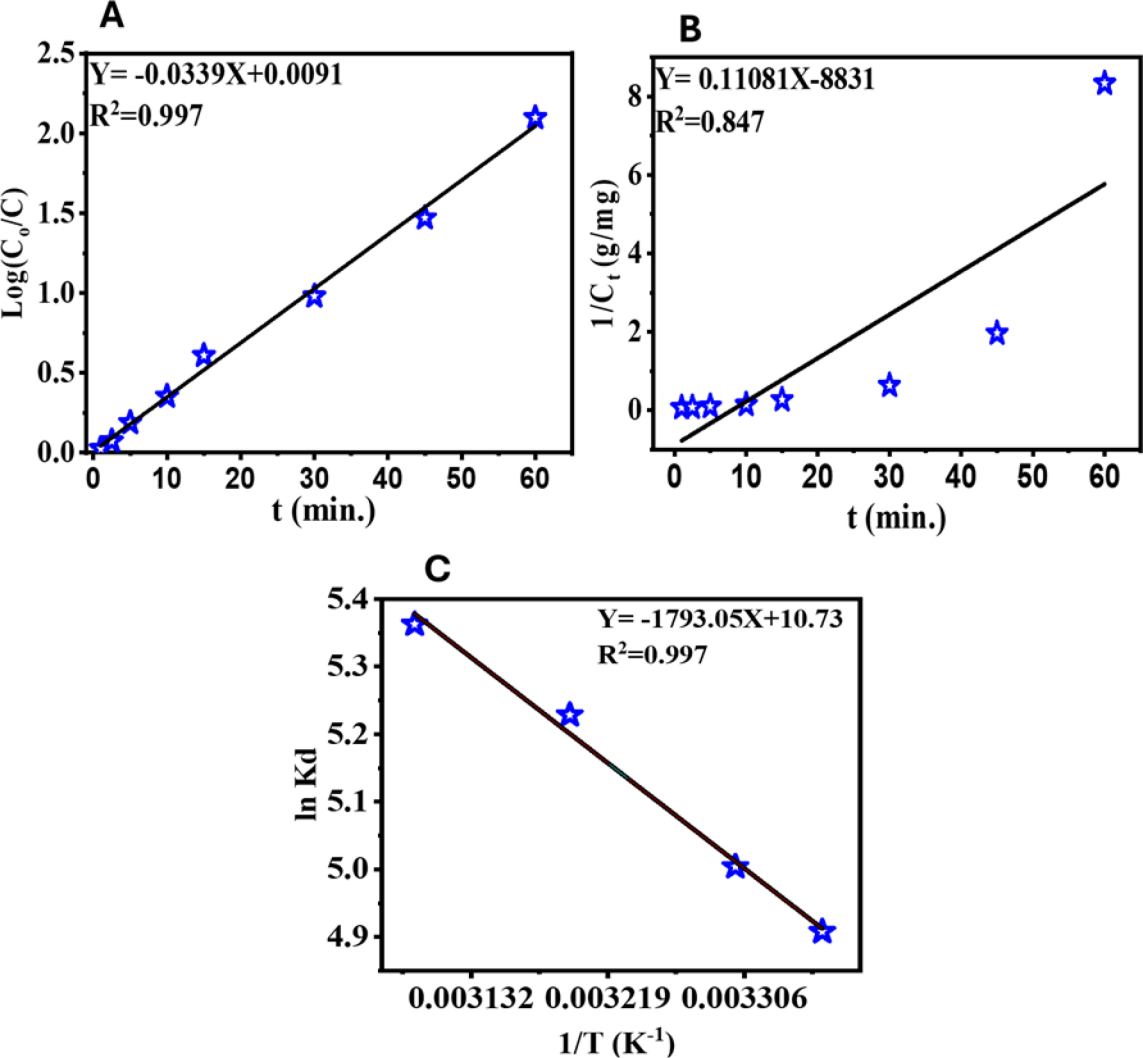
PFO (**A**) and PSO (**B**) kinetic models of CV photocatalytic degradation utilizing HCM; (**C**) Graph of ln K_d_ against 1/T for the determination of the CV-PCD thermodynamic parameters.

Figure 10 (A and B) illustrates the linear correlations of ln (C_o_/C_t_) and 1/C_t_ against irradiation time utilizing the HCM photocatalyst, respectively. The values of K_1_ and K_2_ were computed for PFO & PSO order models and their half-lifetime periods (t_0.5_), and R^2^ are presented in Table 7. The fitting results of PFO (R² = 0.992; RMSE = 0.018) confirm excellent agreement between experimental and theoretical data. The residual analysis revealed a random distribution of errors around zero, confirming that the model is statistically sound. Minor deviations observed at extended reaction times are attributed to partial surface saturation by intermediate species. The PFO rate constant K_1_ was found to be 0.0339 min⁻¹. On the other hand, the PSO model had a lesser correlation value of R^2^ = 0.847. The results indicated that the pseudo-first order (PFO) kinetic model provided a better fit than the pseudo-second order (PSO) model; thus, the PFO model was selected to describe the photocatalytic degradation of CV dye. The strong linearity of the PFO plot further confirms that the degradation rate is directly proportional to the dye concentration, which is a characteristic feature of many photocatalytic systems. The PFO rate constant and half-life (t₀.₅ = 21 min) correspond with the anticipated behavior of photocatalytic systems, indicating a moderate degradation rate that appropriately reflects the kinetics of the process. On the other hand, the half-life period of the PSO model was found to be 60 min. The PSO model, while offering insights into potential surface interactions with HCM, exhibits considerable non-linearity and a suboptimal fit to the experimental data, making it less suitable for characterizing this system. In contrast, the PFO model provides a more precise and mechanistically pertinent characterization of the PCD of CV dye.

### Thermodynamic study

Thermodynamic parameters (standard Gibbs free energy change (ΔG°), standard enthalpy change (ΔH°), and standard entropy change (ΔS°) are significant in determining the feasibility and mechanism of photocatalytic degradation within an isolated system. The energy transfer direction from the aqueous phase to the solid–liquid interface during adsorption as well as degradation of the pollutant indicates these quantities. In this study, thermodynamic behavior for the degradation of CV dye by HCM in the temperature range 298–323 K has been discussed, and values of ΔG°, ΔH°, and ΔS° have been obtained using the following equations: (Eqs. (18 & 19))^67^.

18 $$\:ln{K}_{d}=\frac{{\varDelta\:S}^{o}}{R}-\frac{{\varDelta\:H}^{o}}{RT}$$

19 $$\:{\varDelta\:G}^{o}=\:{\varDelta\:H}^{o}-T{\varDelta\:S}^{o}$$

Here, *T* represents the absolute temperature in (K), *R* is the universal gas constant (8.314 J/mol·K), and *K*_*d*_ denotes the equilibrium constant, which was calculated using the relation K_d_=qe/Ce.

The van’t Hoff plot of ln K_d_ against 1/T (Fig. 10C) demonstrates excellent linearity with R² = 0.997 and RMSE = 0.015, confirming the strong correlation between the experimental data and the theoretical thermodynamic model. Additionally, Table 8 summarizes the values of the thermodynamic parameters for the PCD of CV dye by HCM at various temperatures. The positive enthalpy change (ΔH = + 14.90 kJ/mol) indicates that the PCD of CV dye using HCM is an endothermic process, suggesting that elevated temperatures enhance degradation efficiency^68^. Moreover, this refers to consistent photon absorption that excites electrons from the valence to the conduction band. The positive entropy value (ΔS = + 90.87 J/mol·K) indicates heightened disorder at the solid–liquid interface, attributable to the formation of reactive oxygen species and the degradation of dye molecules into smaller intermediates. The negative Gibbs free energy values (ΔG = − 12.15 to − 14.39 kJ/mol) across all examined temperatures indicate that the photocatalytic breakdown of CV-PCD is spontaneous, driven by photoinduced redox reactions at the catalyst surface^69^. Hence, the rate-determining step is surface-controlled rather than adsorption-limited, which aligns with the photocatalytic mechanism of charge carrier migration and radical formation. Moreover, this process becomes more favorable at higher temperatures, as confirmed by the fact that ΔG becomes increasingly negative with increasing temperature^70,71^. According to our findings, the photocatalytic breakdown of CV dye over HCM is an entropy-driven, spontaneous, endothermic event.

**Table 7:** PFO and PSO kinetic parameters for PCD of CV dye on the surface of HCM.

Pseudo-first order			Pseudo-second order		
R^2^	K_1_ (min^− 1^)	t_0.5_ (min.)	R^2^	K_2_ (g/mg.min)	t_0.5_ (min.)
0.997	0.033	21.004	0.847	0.11	60.6

**Table 8:** Thermodynamic parameters for CV dye removal by HMC at various temperatures.

T, (K)	K_d_	ΔH, (KJ/mol)	ΔS, (KJ/ K.mol)	ΔG, (KJ/mol)
298	4.9	14.90	90.87	− 12.15
303	5.0			− 12.60
313	5.2			− 13.60
323	5.4			− 14.39

### Degradation mechanism

**Fig. 11:**
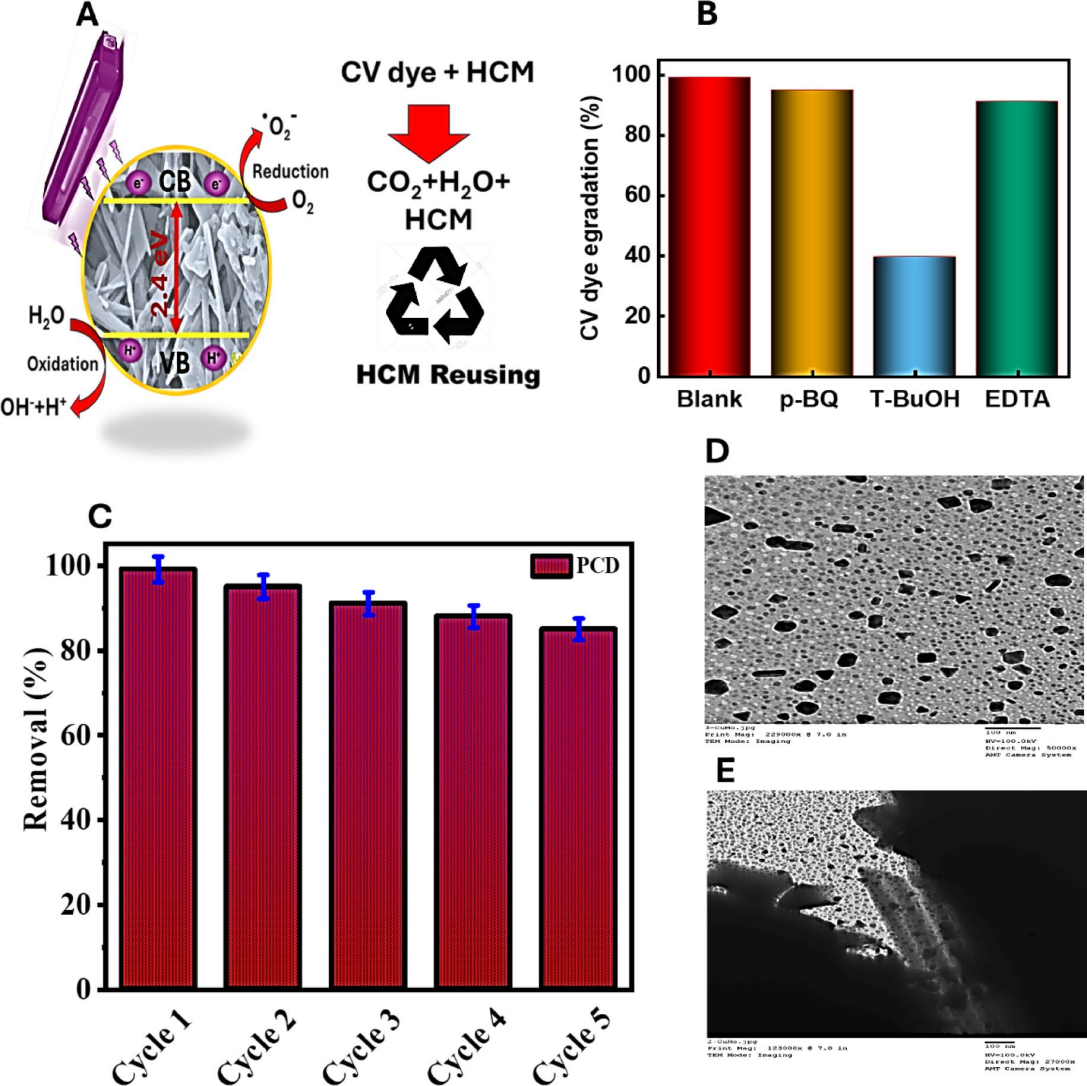
Proposed photo-catalytic mechanism (**A**), Scavenger investigation (**B**), Recycling study of HCM and TEM images of HCM before PCD (**D**) and after (**E**).

Upon exposure to UV radiation, HCM would form e^-^/h ^+^ pairs, as shown in Fig. 11A. The excited state of electrons from the valence band to the conduction band of HCM is demonstrated. The energy gap of HCM has been estimated to be approximately 2.41 eV, allowing for the excitation of electrons by UV radiation. Another effect of this process is the production of reactive oxygen species through redox reactions, which will ultimately lead to the mineralization of the crystal violet dye. The holes created by photocatalysis oxidize water molecules to highly reactive hydroxyl radicals, which play a leading role in the degradation pathways. Meanwhile, the electrons generated by photocatalysis reduce dissolved oxygen (O₂) to superoxide radicals (O₂^·^). Both superoxide species then react with protons in the medium to produce hydroperoxyl radicals (HOO^·^), ultimately converting them to hydrogen peroxide (H₂O₂) and oxygen. The breakdown of H₂O₂ generates extra hydroxyl radicals (^·^OH), hence exacerbating the degradation of CV dye. This series of radical-driven reactions degrades the dye molecules into smaller, environmentally safe compounds. Overall, dye adsorption on the negatively charged HCM surface and the formation of reactive oxygen species under UV light contribute to increased breakdown efficiency. This synergistic effect establishes HCM as an extremely effective photocatalyst for environmental restoration^72^. This synergistic effect establishes HCM as an extremely effective photocatalyst for environmental restoration^72^. On the other hand, experimental explanation was shown as under UV light, H₂O molecules can ngage with holes ($\:{\mathrm{h}\:}_{\mathrm{V}\mathrm{B}}^{+}$) generating OH·, while $\:{\mathrm{e}\:}_{\mathrm{C}\mathrm{B}}^{-}$ can capture O_2_ molecules, producing anionic superoxide ($\:{\mathrm{O}\:}_{2}^{-.}$) radicals at the HCM surface. The ensuing reaction between O₂⁻· and H ^+^ ions yields hydroperoxy (HOO^·^) radicals. Subsequently, the HOO^·^ radical undergoes further transformation into H₂O₂ and O₂. The dissociation of H₂O₂ yields additional OH^·^ radicals, which play a pivotal role in the degradation of CV dye, as exemplified in the subsequent equations^73^:

20 $${\text{HCM }} + {\text{ h}}\upsilon {\text{ }} \to \left( {{\mathrm{e~}}_{{{\mathrm{CB}}}}^{ - } {\text{ + h~}}_{{{\mathrm{VB}}}}^{ + } } \right)@{\mathrm{HCM}}$$

21 $${\text{H2O }} + {\mathrm{h~}}_{{{\mathrm{VB}}}}^{ + } \to {\text{ OH}} + {\text{ H}}^{ + }$$

22 $${\mathrm{O}}_{{\mathrm{2}}} + {\mathrm{e~}}_{{{\mathrm{CB}}}}^{ - } \to {\mathrm{O~}}_{2}^{{ - .}}$$

23 $${\mathrm{O~}}_{2}^{{ - .}} + {\text{ H}}^{ + } \to {\text{ HOO}}$$

24 $${\mathrm{2HOO}}^{.} \to {\mathrm{H}}_{{\mathrm{2}}} {\mathrm{O}}_{{\mathrm{2}}} + {\text{ O}}_{{\mathrm{2}}}$$

25 $${\mathrm{H}}_{{\mathrm{2}}} {\mathrm{O}}_{{\mathrm{2}}} \to {\text{ 2OH}}$$

26 $${\text{CV }} + {\text{ OH}}^{.} \to {\text{ degradation products}}$$

Figure 11B illustrates the effect of various scavengers on the photocatalytic degradation efficiency of crystal violet (CV) dye. The experiments were carried out in the presence of specific scavengers to identify the main reactive species involved in the photocatalytic degradation process. The blank sample (without any scavenger) exhibited the highest degradation efficiency (≈ 99%), demonstrating the excellent photocatalytic performance of the material under standard conditions. Upon the addition of p-benzoquinone (p-BQ), a well-known scavenger for superoxide radicals (·O_2_^-^), a slight decrease in degradation efficiency was observed, suggesting a moderate involvement of superoxide radicals in the reaction. In contrast, the presence of tert-butanol (T-BuOH), a scavenger for hydroxyl radicals (*·OH*), caused a significant drop in degradation efficiency (to around 40%), indicating that hydroxyl radicals are the dominant reactive species responsible for the oxidation of CV dye. When EDTA, a typical hole (h^+^) scavenger and electron donor, was added, the degradation efficiency also decreased, though to a lesser extent than with T-BuOH. This implies that photogenerated holes contribute to the degradation process but play a less significant role than *·OH* radicals. Overall, these results confirm that hydroxyl radicals (·OH) are the primary reactive species in the photocatalytic degradation of crystal violet dye, while superoxide radicals (·O_2_^-^) and photogenerated holes (h^+^) play secondary roles.

### Reusability and stability of HCM

The reusability and structural stability of manufactured photocatalysts are extremely important from an economic standpoint^12,17^. The reusability and structural stability of HCM were investigated by performing five consecutive degradation cycles under identical experimental conditions (Fig. 11C). The photocatalytic efficiency decreased slightly from 99% in the first run to about 91% after the fifth cycle, which can be attributed to minor catalyst loss during recovery and washing. SEM images recorded before and after the degradation tests (Fig. 11 (D and E)) confirmed that the hierarchical plate–rod architecture remained intact, indicating excellent structural stability. Although TOC or COD measurements were not performed, the complete disappearance of the violet crystal absorption band suggests nearly full mineralization. These results demonstrate that the HCM photocatalyst exhibits remarkable stability and reusability for practical environmental applications.

### Biological activity

**Fig. 12:**
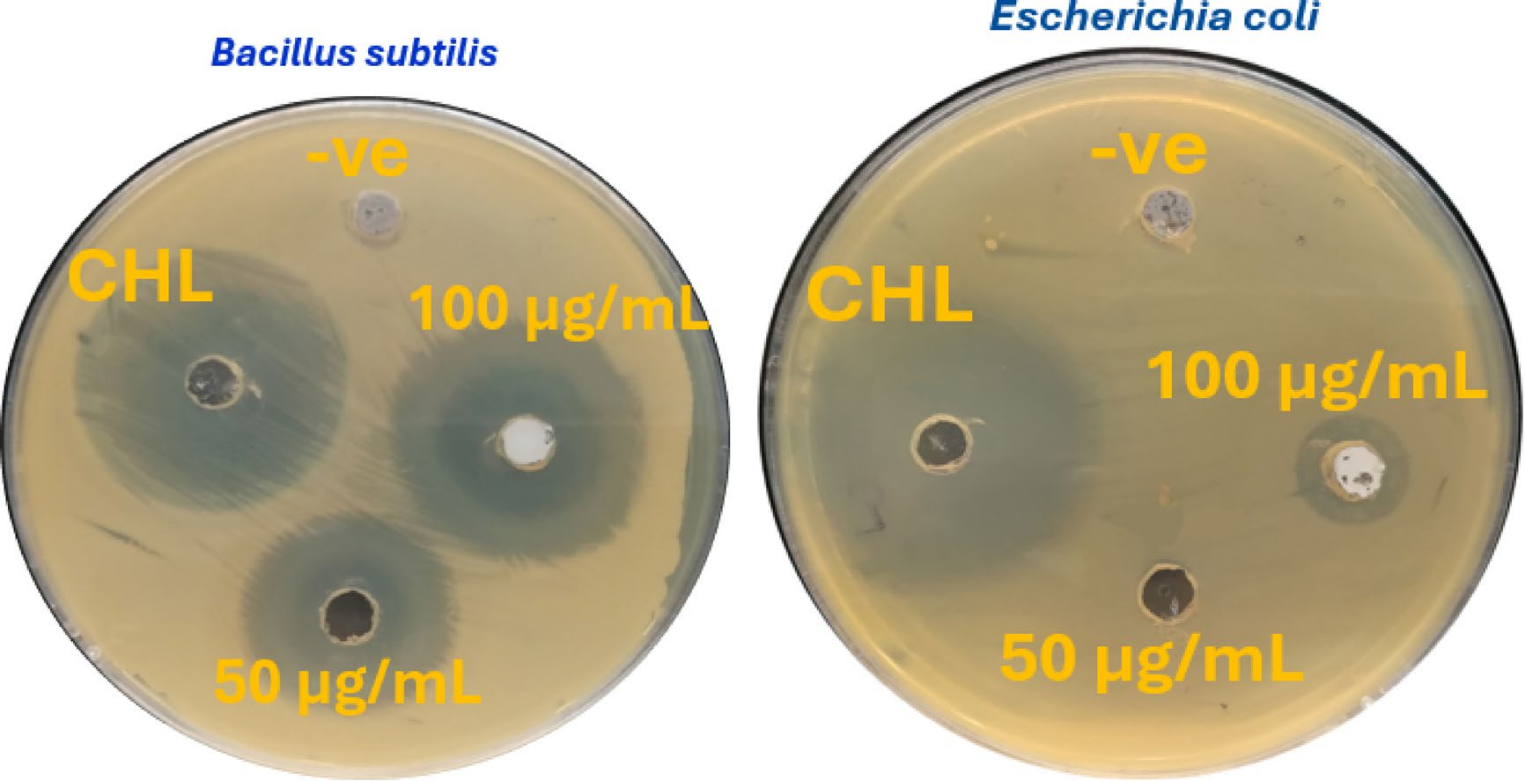
Antimicrobial activity of HCM nanostructure against B. cereus and E. coli.

The synthesized HCM was investigated for their inhibitory effect on the growth of Gram-negative and Gram-positive bacteria: E. coli, P. aeruginosa, B. cereus, and S. aureus. Furthermore, the HCM were tested for their inhibitory effect on the (A) Niger and C. Albian fungi (Table 9**)**. The antimicrobial activity was tested using the disk diffusion method^74^; the clear zone of inhibition around each disk was measured (in mm) and compared to the known sensitive drug, Chloramphenicol (CHL) as an antibacterial drug and Clotrimazole (CLO) as an antifungal drug. In the case of negative control (-ve), the absences of a clear zone indicated that DMSO had no antibacterial or antifungal effect (Fig. 12). The HCM showed varying antimicrobial action depending on the microorganism species and the compound itself. HCM proved to be an excellent candidate as antibacterial agent, being able to inhibit some bacterial species. P. aeruginosa, (B) cereus and S. aureus were the most sensitive to HCM. E. coli bacteria did not show any sensitivity to HCM at a concentration of 50 µg/mL, while when the concentration increased to 100 µg/mL, a sensitivity within the inhibition zone, at 11.20 mm, appeared. Figure 12 shows that the concentration of the HCM nanostructure increases from 50 to 100 µg/mL, the antibacterial and antifungal activities also increase. In case of antifungal activity, HCM showed good antifungal results against A. Niger, and (C) Albian within the inhibition zone, at 20.11, 19.02 mm, respectively, which are close to those of the standard Clotrimazole.

Tanasic et al.^75^ proposed a plausible mechanism for the antimicrobial activity of HCM nanoparticles against fungi, suggesting that their action is associated with alterations in the structure and function of fungal cells. These nanoparticles can induce cell death by interacting with cellular DNA, thereby interfering with replication and transcription processes. Furthermore, HCM may inactivate key enzymes by binding to their sulfhydryl (–SH) groups, leading to inhibition of metabolic activity and growth arrest. Another hypothesis indicates that HCM-induced oxidative stress within microbial cells contributes significantly to cell damage and eventual death^76^. Nevertheless, further investigations are required to elucidate the detailed molecular mechanisms underlying HCM-mediated inhibition of microbial proliferation.

**Table 9:** Antibacterial activity of HCM nanostructure (Inhibition zone in (mm), and mean ± standard deviation; *n* = 3).

Nanoparticle	Volume (µg/mL)	Microorganisms					
		Gram-negative bacteria		Gram-positive bacteria		Fungi	
		E. coli	*P*. aeruginosa	B. cereus	S. aureus	A. Niger	C. albicans
HCM	50	0	15.33 ± 0.17	18.34 ± 0.15	16.12 ± 0.05	15.32 ± 0.16	13.31 ± 0.16
	100	11.20 ± 0.15	17.41 ± 0.25	20.44 ± 0.07	20.25 ± 0.13	20.11 ± 0.16	19.02 ± 0.16
CHL	100	23.41 ± 0.21	20.21 ± 0.06	23.51 ± 0.16	21.30 ± 0.14	–	–
CLO	100	–	–	–	–	22.02 ± 0.16	20.45 ± 0.16

## Conclusion

HCM nanostructure was successfully synthesized using the hydrothermal method and then characterized using different techniques such as XRD, SEM, TEM, IR, and EDX mapping to confirm its constituents and its nanocrystalline structure. The synthesized HCM exhibits a suitable band gap (~ 2.4 eV) for photocatalysis. The photocatalytic degradation of crystal violet (CV) dye was optimized using response surface methodology, with pH, irradiation time, and catalyst dosage identified as the key parameters. Under optimal conditions (pH 10, 60 min, 15 mg/L CV, and 10 mg catalyst dosage), nearly complete degradation of more than 99% was achieved. Kinetic studies followed a pseudo-first-order model, while thermodynamic analysis indicated that the process is spontaneous, endothermic, and entropy-driven. Mechanistic insights confirmed the role of reactive oxygen species in the degradation pathway. Furthermore, the material exhibited notable antimicrobial activity, highlighting its potential as an efficient, low-cost, and multifunctional candidate for environmental remediation.

## Data Availability

All data generated or analyzed during this study are included in this article, and the raw data is available from the corresponding author if it is requested.
